# Endophytic *Trichoderma asperellum* WNZ-21 produces novel bioactives with anticancer, anti-inflammatory, and hemoprotective properties from fermented bean biomass

**DOI:** 10.3389/fmicb.2025.1609361

**Published:** 2025-06-10

**Authors:** Yasmene F. Alanazi, Salma Saleh Alrdahe, Zeiad Moussa, Doaa Bahaa Eldin Darwish, Haifa Alrdahi, WesamEldin I. A. Saber

**Affiliations:** ^1^Department of Biochemistry, Faculty of Science, University of Tabuk, Tabuk, Saudi Arabia; ^2^Department of Biology, Faculty of Science, University of Tabuk, Tabuk, Saudi Arabia; ^3^Microbial Activity Unit, Department of Microbiology, Soils, Water and Environment Research Institute, Agricultural Research Center, Giza, Egypt; ^4^Department of Computer Science, Faculty of Science and Engineering, University of Manchester, Manchester, United Kingdom

**Keywords:** common bean, semi-solid-state fermentation, *Trichoderma* sp., amino acids, multibiological activity

## Abstract

The increasing demand for novel bioactive compounds is driving research into natural sources, particularly through the valorization of agricultural residues. Endophytic fungi have emerged as a promising source of bioactive metabolites with diverse therapeutic potential. We employed a novel approach utilizing the isolated *Trichoderma asperellum* WNZ-21 [identified morphologically and molecularly (ITS: OR857252, *tef1* gene: PP069312)] in semi-solid-state fermentation to bioconvert common bean biomass residue (RCBB) into a rich source of metabolites with significant therapeutic potential. First, *T. asperellum* efficiently degraded 67.19% RCBB, exhibiting robust enzymatic activity (cellulase: 18.067 units per gram (U/g), xylanase: 15.967 U/g, protease: 5.550 U/g). The fermented RCBB filtrate exhibited a rich chemical profile, including (mg/g RCBB) amino acids (13.51), phenolics (55.22), flavonoids (11.22), tannins (18.90), and several secondary metabolites. FT-IR analysis indicated the presence of a complex mixture of amino acids, polysaccharides, proteins, and other metabolites. GC-MS analysis identified 17 compounds. The fermented biomass possesses multi-biological activities, including anticancer against hepatocellular carcinoma (IC_50_ = 35.62 μg/mL) and mammary gland breast cancer (IC_50_ = 59.20 μg/mL) cell lines, with reduced toxicity toward normal human lung fibroblast cells (IC_50_ = 76.33 μg/mL). It also exhibits anti-hemolytic activity in erythrocyte protection assays, reducing oxidative stress-induced hemolysis to 4.40%. Additionally, the filtrate demonstrates anti-inflammatory activity in a carrageenan-induced paw edema model in rats, providing 36.50% protection after 5 h of treatment. Our findings establish RCBB valorization as a promising route to produce multi-bioactive fungal metabolites. Future research should focus on isolating active compounds, optimizing production, and conducting *in vivo* studies to fully realize therapeutic potential.

## 1 Introduction

The ever-increasing generation of plant biomass residues demands innovative solutions, not only for sustainable resource management but also for the discovery of novel drugs with diverse therapeutic properties (El-Naggar et al., 2014). In the face of growing antibiotic resistance and chronic illnesses, researchers are actively exploring natural sources for new drugs. Among these, endophytic fungi have arisen as an auspicious resource due to their bioactive metabolites with significant therapeutic potential (De Silva et al., 2019; Sridharan et al., 2021). These fungi, which reside within plant tissues, possess a unique enzymatic arsenal capable of degrading complex organic matter, making them ideal candidates for the valorization of agricultural residues.

Millions of tons of plant protein residues are generated annually, and their disposal poses significant environmental and economic challenges. Traditional methods of biomass degradation, such as acid hydrolysis, are often inefficient and can lead to the destruction of valuable compounds, including amino acids (Moussa et al., 2023). In contrast, microbial degradation, particularly through semi-solid-state fermentation (SSSF), offers a sustainable and efficient alternative for converting these residues into bioactive metabolites. This less commonly employed technique falls between solid and liquid-state fermentation, and provides several advantages, including reduced energy requirements, increased productivity, and higher concentrations of target molecules (El-Metwally et al., 2023; Elsayed et al., 2021).

*Phaseolus vulgaris* L. (common bean), a key global crop, which accounts for 85% of the world’s bean production, generates substantial biomass residues rich in protein content (Nasar et al., 2023). The residue of common bean biomass (RCBB) is recalcitrant and poses challenges for biotechnological applications. However, they represent an untapped resource to produce bioactive compounds through microbial fermentation (Al-Askar et al., 2022; Moussa et al., 2023). While RCBB can be composted for traditional uses, its potential as a basis of cost-effective multi-bioactive metabolites for medical applications remains largely unexplored.

This study addresses this gap by exploring the potential of endophytic fungi to valorize RCBB through SSSF. Endophytic fungi, particularly those belonging to *Trichoderma* spp., are known to produce a diverse array of enzymes and secondary metabolites with significant bioactivities (Al-Askar et al., 2022; De Silva et al., 2019). By leveraging the enzymatic capabilities of these fungi, we aim to transform RCBB into valuable bioactive compounds with potential therapeutic applications.

Recent advances in microbial biomass valorization have highlighted the potential of endophytic fungi to produce bioactive metabolites from agricultural residues. For instance, studies have demonstrated the ability of *Trichoderma* species to degrade lignocellulosic biomass and synthesize enzymes such as cellulase, xylanase, and protease, which are essential for breaking down complex plant materials (Al-Askar et al., 2022; Moussa et al., 2023). Additionally, the use of untargeted metabolomics and bioassay-guided fractionation has enabled the identification of novel bioactive compounds from fungal filtrates, further underscoring the potential of these microorganisms in drug discovery (Elattar et al., 2024; Salem et al., 2022; Wang et al., 2023).

Despite advancements in bioconversion, a significant gap exists in the direct utilization of proteinaceous residues such as RCBB by endophytic fungi for the synthesis of bioactive metabolites. To address this gap and provide a sustainable waste valorization strategy, this study aimed to identify and select an endophytic fungus with potent enzymatic activity for the efficient bioconversion of RCBB via a less common technique (semi-solid-state fermentation). Following the selection of *Trichoderma asperellum* WNZ-21 based on its superior hydrolytic capabilities, the next novel step was to produce and comprehensively profile the multi-biological activity of the resulting fungal filtrate, including the identification of various bioactive compounds, and to evaluate its potential for anticancer, antihemolytic, and anti-inflammatory applications. This novel integrated approach bridges two interconnected research areas: (1) Trichoderma’s capacity to degrade plant waste through, and (2) the multi-bioactive potential of the resulting metabolite-rich fermentation filtrate.

## 2 Materials and methods

### 2.1 Isolation of the endophytic fungi

The endophytic fungi were isolated from common bean plants. Plant samples were collected from different locations in the Tabuk region, Saudi Arabia, from an area of 50 × 50 m around each sampling site in a random zigzag pattern. The surveyed area lies between latitudes of 28°23′59″ N, and longitudes of 36°34′17″ E, and elevation above sea level is 768 m. The collected plants were kept in polyethylene bags at 4°C and transferred to the laboratory for the isolation process using the method reported by Kuo et al. (2021) with some modifications.

Leaves and stems were washed with tap water to remove surface particles. The epiphytic microbes were removed through surface sterilization by immersing in ethyl alcohol (70%/10 s), then soaking in sodium hypochlorite (4%/30 s), before washing twice in sterile distilled water. Plants were cut into pieces 0.5–1.0 cm long. The segments were placed on 2% malt extract agar (BD Biosciences, Franklin Lakes, NJ, United States) containing 0.5 g/L penicillin G and streptomycin sulfate (Sigma-Aldrich, St. Louis, MO, United States), at 5 mg/L each. The inoculated plates were incubated at 25 ± 2°C under an alternating 12 h light/darkness cycle of cool white, fluorescent light. The developing fungal colonies were observed daily for 1 month using both stereo and compound microscopes to aid in the identification of the recovered fungi. Fungi with different mycelial morphotypes were isolated using single-spore and/or hyphal-tip techniques and subcultured on malt extract agar. The purified isolates were preserved on potato dextrose agar plates (BD Difco, Becton, Dickinson and Company, United States).

### 2.2 Fungal identification

#### 2.2.1 Morphological identification

All fungi were morphologically identified according to their fungal morphology, cultural features, and microscopic characteristics. Four fungal genera were specified. The general morphological characteristics of *Trichoderma* spp. were performed with the aid of Asis et al. (2021) and Siddiquee and Siddiquee (2017). *Aspergillus* species were identified according to Diba et al. (2007), Domsch et al. (1980), Samson and Pitt (2000). General characteristics of *Penicillium* spp. were identified compared with (de Hoog et al., 2005; Samson et al., 2014; St-Germain and Summerbell, 2003; Sutton et al., 1998). Morphological characteristics of the genus *Mucor* spp. were identified according to de Hoog et al. (2005) and Sutton et al. (1998).

#### 2.2.2. Scanning electron microscopy

Fungal mycelium was fixed with 2% (*w/v*) osmium tetroxide vapor for 20 h, dehydrated through a graded ethanol series (30 –100%), and critical point dried using liquid CO_2_. Samples were mounted on copper stubs, sputter-coated with gold-palladium (15 nm thickness), and imaged using a JEOL JSM-7600F scanning electron microscope at 10 kV (Bozzola and Russell, 1999; Goldstein et al., 2017).

#### 2.2.3 Molecular identification (ITS and *tef1* gene sequencing)

The isolation and purification of genomic DNA from the selected fungus were performed using Norgen’s Plant/Fungi DNA Isolation Kit (Norgen Biotek Corp., Thorold, ON, Canada). The study focused on amplifying two specific regions: the internal transcribed spacer (ITS) region and the translation elongation factor 1-alpha (*tef1*) gene. The primer pairs used were ITS-1 F (5′-TCCGTAGGTGAACCTGCGG-3′)/ITS-4 R (5′-TCCTCCGCTTATTGATATGC-3′) for amplifying the ITS region (∼ 600 bp) and tef1-F (5′-CATCGAGAAGTTCGAGAAGG-3′)/tef1-R (5′-GCCATCCTTGGGAGATACCAGC-3′) for amplifying the tef1 gene (∼730 bp) (Cai and Druzhinina, 2021; White et al., 1990).

The PCR was conducted in a 50 μL mixture, comprising 25 μL of Master Mix (Sigma), 3 μL of each primer (10 pmol/μL), 3 μL of template DNA (10 ng/μL), and 16 μL of distilled water. Thermal cycling conditions consisted of an initial denaturation step at 94°C for 5 min, followed by 40 cycles of denaturation at 94°C for 40 s, annealing at 50°C for 1 min, and extension at 72°C for 1 min, with a final step at 72°C for 7 min. The PCR products underwent electrophoresis on a 1.5% agarose gel stained with ethidium bromide (0.5 μg/mL) in 1X TBE buffer at 95 V for 45 min, followed by visualization under UV light. A 100-nucleotide DNA ladder (Thermo Scientific, Germany) served as the molecular size standard. Subsequently, the PCR products were purified using an EZ-10 spin column PCR purification kit (Bio Basic Inc., Ontario, Canada). The PCR segments were sequenced bidirectionally using the same PCR amplification primers, and sequenced. The sequence was deposited in the GenBank of the National Center for Biotechnology Information after being aligned and compared to the database using the BLASTn algorithm tool.^1^ The evolutionary analyses of the nucleotide sequences were performed using MEGA11. For each sequence pair, the ambiguous positions were eliminated and inferred using the Neighbor-Joining technique (Saitou and Nei, 1987). Jukes-Cantor distances (Jukes and Cantor, 1969) were employed, and the optimal tree was presented with branch lengths reflecting evolutionary units and bootstrap support values (1000 replicates) *(Felsenstein, 1985)*.

### 2.3 Fungal fermentation of RCBB

The previously collected common bean plants were used to prepare RCBB before being used as a fermentation substrate during SSSF. The RCBB was dried (70°C) overnight and ground into pieces, measuring 1–2 mm. The chemical composition of RCBB was carried out according to AOAC (2019).

The procedure of SSSF was carried out in 250 mL Erlenmeyer flasks. The RCBB-based medium was supported with 10 mL salt solution (composed of NaH_2_PO_4_; 12.8, KH_2_PO_4_; 3, NaCl; 0.5, MgSO_4_.7H_2_O; 0.5, NH_4_Cl; 1.0, and CaCl_2_.2H_2_O; 0.01 g/L, pH 6) (Tunga et al., 1998) per 1 g RCBB. The media were autoclaved (15 min/121°C) before being inoculated with 1 ml of 10∧6 spore suspension (from the 7-day-old culture) and incubated in a CGI-400P growth chamber (Taisite Lab.) under cool white, fluorescent light with a 12-hour light/darkness cycle (28 ± 2°C, 65% humidity, and illumination set to 4,000 lux.). A medium without inoculation was used as a control. After 10 days of incubation, 10 mL of Tween 80 (0.01%) was added, and the flasks were shaken (30 min/150 rpm), then filtered and centrifuged (3,354 g/20 min) to separate the fungal filtrate. The residual RCBB was weighted to calculate the remaining biomass (%).

### 2.4 Biochemical analysis of the fermented fungal filtrate

#### 2.4.1 Protease assay and total free amino acids content

The proteolytic activity and TFAAs were determined, utilizing casein as a substrate and Folin-Ciocalteu reagent (Fluka, Biochemical Inc., Romania). Tyrosine was used as a standard (Cupp-Enyard, 2008). One unit (U) of protease is defined as the quantity of enzyme that liberates 1 μmol of tyrosine/gram/min (1 μmol/g/min) under the given conditions.

#### 2.4.2 Cellulase assay

Cellulase activity was determined using microcrystalline cellulose as the substrate. Briefly, the reaction mixture contained 1.5 mL of 1% (*w/v*) microcrystalline cellulose in 0.05 M sodium citrate buffer (pH 4.8) and 0.5 mL of fungal filtrate. The mixture was incubated at 50°C for 1 h (Saber et al., 2015). Next, the released reducing sugars were quantified using the 3,5-dinitrosalicylic acid (DNS) method (Miller, 1959). Glucose was used as the standard for calibration. One cellulase U was defined as the enzyme amount required to release one μmol of glucose/min/g RCBB under specified assay conditions.

#### 2.4.3 Xylanase assay

Xylanase activity was measured using 1% (*w/v*) oat spelt xylan (Sigma-Aldrich). Briefly, the reaction mixture comprised 0.5 mL of fungal filtrate and 1.5 mL of substrate solution in 0.1 M phosphate buffer (pH 6.0), then incubated at 50°C for 15 min (Abdelwahed et al., 2011). The released reducing sugars were quantified via the DNS method (Miller, 1959), with xylose (Sigma-Aldrich) as the standard. One xylanase U was defined as the enzyme quantity required to release 1 μmol of xylose/min/g RCBB under the assay conditions.

#### 2.4.4 High-performance liquid chromatography

The amino acids (AAs) profile of the fungal filtrate was detected with an Agilent 1260 series HPLC system equipped with an Eclipse Plus C18 column (4.6 × 250 mm i.d., 5 μm). A mobile phase composed of sodium phosphate dibasic/sodium borate buffer (pH 8.2), acetonitrile, methanol, and water (45:45:10 *v/v/v*) was employed at a flow rate of 1.5 mL/min. The filtrate (1.25 mL) was acid hydrolyzed by mixing with 1.25 mL of water and 2.5 mL of 6 M HCl. The resulting mixture was incubated at 100°C for 24 h and subsequently filtered. One milliliter of the processed filtrate was injected into the HPLC system for analysis (Jajić et al., 2013). Identification of AAs was achieved by comparing retention times of the calibration curve generated from a standard under identical conditions.

#### 2.4.5 Gas chromatography-mass spectrometry analysis

The lyophilized fungal filtrate was resuspended in methoxyamine hydrochloride (20 mg/mL pyridine) for oximation (90 min). Bis(trimethylsilyl)trifluoroacetamide with 1% trimethylchlorosilane was then added for derivatization (70 °C, 30 min) to TMS derivatives suitable for GC-MS. An Agilent Technologies 7890B GC coupled to a 5977A MS was used for analysis. The HP-5MS column (30 m × 0.25 mm × 0.25 μm) with the carrier gas (hydrogen, 1.0 mL/min) and a 2 μL injection volume separated the metabolites. The oven program started at 50°C and ramped to 300°C at 10°C/min. The detector and injector temperatures were kept at 250°C. Electron ionization (70 eV) generated mass spectra (m/z 30–700) after a 9-min solvent delay. The temperatures of the mass and transfer line were 230 and 150°C, respectively. Metabolite identification utilized the Wiley and NIST Mass Spectral Libraries.

#### 2.4.6 Phytochemical content in the fungal filtrate

The photochemical content in the fungal filtrate, resulting from the degradation of plant biomass, was determined. The total of phenolics was measured using the Folin-Ciocalteu reagent, using gallic acid as a standard (Sánchez-Rangel et al., 2013). The total flavonoids were measured, applying catechin as a standard (Zhishen et al., 1999). The tannin content was measured using the vanillin-hydrochloride method (Aberoumand, 2009; Burlingame, 2000). Tannic acid was utilized as a standard to determine the total tannin content.

#### 2.4.7 Fourier transform infrared spectroscopy

The fungal filtrate (5 mg) was mixed with 100 mg potassium bromide, and the mixture was analyzed using a Bruker Vertex 70 RAM II FT-IR spectrometer. Spectra were recorded in the 4,000–500^–1^ range at 4 cm^–1^, with 32 scans averaged per sample. Peaks were analyzed for functional groups to characterize the chemical composition.

### 2.5 Biological activity of the fermented fungal filtrate

#### 2.5.1 Cytotoxicity and anticancer tests

The MTT (3-[4,5-dimethylthiazol-2-yl]-2,5-diphenyl tetrazolium bromide) assay is largely used for measuring cell viability and cytotoxicity of a compound. Normal (human lung fibroblast; WI38) and tumor (hepatocellular carcinoma; HePG2, and mammary gland breast cancer; MCF7) cell lines were obtained from ATCC through VACSERA, Cairo, Egypt. Doxorubicin served as the standard chemotherapeutic anticancer (control). Cells were cultured in RPMI-1640 medium (Sigma Co., Louis, MO, United States) supplemented with fetal bovine serum (10%) (Thermo Fisher Scientific, GIBCO, United Kingdom), streptomycin (100 μg/mL), and penicillin (100 units/mL) under 5% CO_2_ conditions at 37°C. After seeding individually in a 96-well plate at a density of 10^4^ cells/well, the cells were incubated (48 h/37°C with 5% CO_2_), then exposed to various concentrations (1.56–100 μg/mL) of the lyophilized fungal filtrate for an additional 24 h. Next, 20 μL (5 mg/mL) of MTT was mixed and incubated for 4 h. Hundred microliter of dimethyl sulfoxide was added to the wells to dissolve purple formazan (Denizot and Lang, 1986). The absorbance (*A* at λ = 570 nm) of the developed color intensity was measured utilizing a plate reader (EXL 800, Cranston, RI, United States). Relative cell viability was calculated (Equation 1), and the IC_50_ against the cell line was determined.


(1) $$\mathrm{Cell}\,\mathrm{viability}\%=\left(A_{570}\,\mathrm{sample}/A_{570}\,\mathrm{untreated}\,\mathrm{control}\right)\times 100$$

#### 2.5.2 Animal studies

Male Wistar rats (6 weeks old, 164–176 g), gifted from the Animal House of the National Research Center, Cairo, Egypt, were acclimatized for 1 week under standard laboratory conditions. Animals were provided with standard rodent chow and tap water *ad libitum* and kept on a 12 h light/12 h dark cycle at 24 ± 1°C and humidity of 50 ± 10%.

##### 2.5.2.1 Anti-hemolytic assay

The erythrocyte hemolysis assay was used to assess the antihemolytic activity of the fungal filtrate to protect red blood cells (RBCs) from lysis induced by oxidative stress. The blood samples were collected into heparinized tubes from rats (*n = 6* per group) through cardiac puncture (Yang et al., 2005). Erythrocytes were isolated from the plasma by centrifugation. The buffy coat was then washed thoroughly 3 times with sterile saline (0.89% *w/v* NaCl, pyrogen-free) to eliminate residual plasma. The washed erythrocytes were centrifuged at 834 *g* for 10 min to obtain a standardized erythrocyte suspension. The fungal filtrate (5 mg/mL) was added to a 10% erythrocyte suspension in phosphate-buffered saline (pH 7.4) and incubated for 45 min. at 37°C. A control sample containing L-ascorbic acid was incubated with the erythrocyte suspension without the fungal filtrate. Distilled water served as the maximal hemolytic control. Following incubation, erythrocytes were separated by centrifugation. Hemoglobin release, indicative of lysed RBCs, was quantified at 540 nm. The percent hemolysis was calculated by subtracting the value obtained from the saline control-treated group. The hemolysis percentage was calculated using Equation (2):


(2) $$\mathrm{Hemolysis}\left(\%\right)=\left(\frac{\mathrm{Absorbance}\,\left(\mathrm{sample}\right)}{\mathrm{Absorbance}\,\left(\mathrm{control}\right)}\right)\times 100$$

##### 2.5.2.2 Anti-inflammatory assay

The anti-inflammatory activity of the fungal filtrate was appraised *in vivo* by applying carrageenan-induced paw edema in a rat model (Morris, 2003). Briefly, carrageenan, a polysaccharide derived from red seaweed (1% in sterile saline, Sigma Co., United States), was prepared by heating to 90°C without boiling and cooling to room temperature. Rats (18 individuals) were randomly assigned to three groups (*n* = 6/group) using a blinded randomization procedure to minimize bias. The right hind paw of each rat received a sub-plantar injection of carrageenan solution (0.1 mL). The treatment group received an intraperitoneal injection of the fungal filtrate (5 mg/mL), while the positive control group received indomethacin (a nonsteroidal anti-inflammatory drug, NSAID) at 5 mg/kg. Paw thickness was measured using a vernier caliper at baseline and 0.5, 1, 2-, 3-, 4-, and 5 h post-injection. Paw edema was calculated for each rat by subtracting the baseline paw thickness from the thickness measured at each time point. The anti-inflammatory protection (reduction in paw edema (swelling) induced by carrageenan) was calculated (Equation 3):


(3) Antiinflammatory(protection,%)=

$$\left(1-\left(\frac{\mathrm{Mean}\,\mathrm{of}\,\mathrm{the}\,\mathrm{treated}\,\mathrm{group}}{\mathrm{Mean}\,\mathrm{of}\,\mathrm{the}\,\mathrm{control}\,\mathrm{group}}\right)\right)\times 100$$

No euthanasia was used. All protocols complied with the guidelines and regulations of the Care and Use of Laboratory Animals published by the US National Institutes of Health (NIH publication no.85-23, revised 1996) (Morris, 2003). The study followed the guidelines of Animal Research: Reporting of In Vivo Experiments (ARRIVE).^2^

### 2.6 Data analysis

CoStat (version 6.45, CoHort Software, Birmingham, UK) software was utilized. One-way ANOVA of a completely randomized design was applied to analyze the data mean (± standard deviation, SD). Tukey’s HSD (Honestly Significant Difference) post-hoc test (α ≤ 0.05) was used for pairwise comparisons of means, applying minimum significant difference.

## 3 Results

### 3.1 The recovered fungal isolates

**FIGURE 1:**
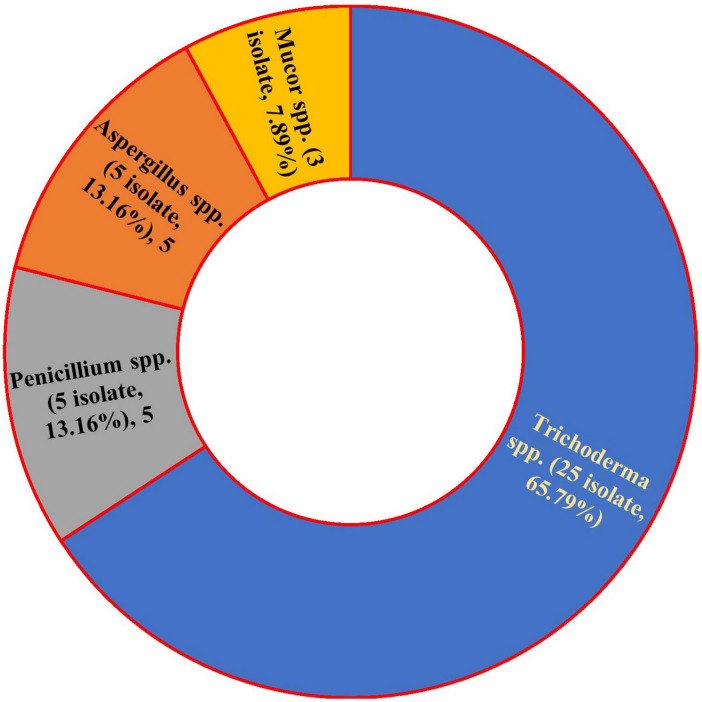
Prevalence and occurrence of common bean endophytic fungi.

This study isolated 38 endophytic fungi from common bean plants (Figure 1). The fungal isolates were initially identified to belong to four genera: *Trichoderma* spp. (25 species, 65.8%), *Aspergillus* spp. (5 species, 13.2%), *Penicillium* spp. (5 species, 13.2%), and *Mucor* (3 species, 7.9%). *Trichoderma* spp. exhibited the highest prevalence among the isolated endophytes. The macroscopic and microscopic features of the four isolated fungal species were determined (Supplementary File).

### 3.2 Screening the hydrolytic activity

First, the chemical composition of RCBB consists of 90.0% dry matter, with a 6.42% ash content. The organic matter content is 85.58%, which includes 15.80% crude protein and 37.50% crude fiber. The ether extract is 1.520%. The organic carbon percentage is calculated to be 38.51%, and the total nitrogen (N) content is 2.53%. This results in a C/N ratio of 15.22, indicating the balance of carbon and nitrogen in RCBB.

The ability of the endophytic fungi to degrade RCBB using SSSF (Table 1 and Supplementary Figure 1) was tested. Among the fungi studied, *Trichoderma* sp. WNZ-21 was selected as a notably robust profile of hydrolytic enzymes, including cellulase (18.067 U), xylanase (15.967 U), and protease (5.550 U), and yielded the highest levels of TFAAs (12041.97 μg/g RCBB), achieving the greatest biomass degradation (32.81 ± 0.65% remaining biomass) compared to others.

**TABLE 1:** Hydrolytic potentiality, total free amino acids production, and the remaining biomass as a result of the fungal activity on semi-solid-state fermentation medium containing the residue of common bean biomass as substrate.

Fungus		Hydrolytic enzyme (U)			Total free amino acids (μg/g)	Remaining biomass, %
		Cellulase	Xylanase	Protease		
*Trichoderma* sp. 1		12.97 ± 0.15	14.58 ± 0.88	3.72 ± 0.20	6454.62 ± 35.70	63.47 ± 0.56
*Trichoderma* sp. 2		12.37 ± 0.15	13.55 ± 2.81	3.38 ± 0.21	6054.95 ± 47.60	65.38 ± 1.14
*Trichoderma* sp. 3		12.18 ± 0.15	13.30 ± 0.81	3.88 ± 0.22	8652.78 ± 39.27	52.30 ± 1.47
*Trichoderma* sp. 4		12.85 ± 0.15	15.08 ± 1.91	4.22 ± 0.23	8852.62 ± 38.08	50.90 ± 0.95
*Trichoderma* sp. *5*		10.07 ± 0.14	12.77 ± 0.77	4.05 ± 0.24	9252.28 ± 21.55	47.39 ± 1.20
*Trichoderma* sp. 6		12.52 ± 0.14	12.58 ± 0.76	3.72 ± 0.25	6854.28 ± 19.04	62.37 ± 1.41
*Trichoderma* sp. 7		13.92 ± 0.14	14.52 ± 0.88	5.05 ± 0.26	8652.78 ± 35.70	52.30 ± 1.47
*Trichoderma sp.* 8		10.10 ± 0.14	13.08 ± 0.79	4.05 ± 0.27	8053.28 ± 7.14	54.83 ± 0.01
*Trichoderma* sp. 9		12.27 ± 0.14	13.27 ± 2.80	4.38 ± 0.28	8452.95 ± 45.22	52.58 ± 0.01
*Trichoderma* sp. 10		12.35 ± 0.13	13.15 ± 0.80	4.38 ± 0.29	7253.95 ± 26.18	59.21 ± 0.18
*Trichoderma* sp. 11		12.05 ± 0.13	13.57 ± 0.82	4.38 ± 0.30	6854.28 ± 12.50	62.03 ± 0.84
*Trichoderma* sp. 12		13.53 ± 0.13	13.50 ± 3.82	4.72 ± 0.31	6654.45 ± 32.13	63.12 ± 0.76
*Trichoderma* sp. 13		13.73 ± 0.13	15.35 ± 2.93	4.38 ± 0.32	8253.12 ± 24.63	53.47 ± 0.41
*Trichoderma* sp. 14		12.85 ± 0.13	13.82 ± 0.84	3.38 ± 0.33	8253.12 ± 27.65	53.70 ± 0.01
*Trichoderma* sp. 15		12.35 ± 0.12	13.43 ± 0.81	3.88 ± 0.34	8452.95 ± 28.71	52.58 ± 0.01
*Trichoderma* sp. 16		15.87 ± 0.12	15.58 ± 0.94	3.88 ± 0.35	8952.53 ± 29.76	50.18 ± 0.71
*Trichoderma* sp. 17		14.90 ± 0.12	14.35 ± 0.87	3.55 ± 0.36	7453.78 ± 30.82	58.18 ± 0.01
*Trichoderma* sp. 18		11.92 ± 0.12	13.00 ± 0.79	3.72 ± 0.37	8053.28 ± 31.88	54.82 ± 0.01
*Trichoderma* sp. 19		13.80 ± 0.11	14.23 ± 0.86	5.38 ± 0.38	7853.45 ± 32.94	56.30 ± 0.61
*Trichoderma* sp. 20		15.50 ± 0.11	17.18 ± 1.04	4.22 ± 0.39	9072.43 ± 33.99	49.11 ± 0.01
*Trichoderma* sp. 21		18.07 ± 0.11	15.97 ± 0.97	5.55 ± 0.40	12041.97 ± 35.05	32.81 ± 1.05
*Trichoderma* sp. 22		13.65 ± 0.11	14.92 ± 0.90	4.55 ± 0.26	8053.28 ± 89.26	56.61 ± 1.63
*Trichoderma* sp. 23		13.10 ± 0.11	13.03 ± 0.79	4.22 ± 0.28	7054.12 ± 41.38	60.44 ± 0.01
*Trichoderma* sp. 24		13.13 ± 0.10	13.92 ± 0.84	5.22 ± 0.29	7253.95 ± 13.09	58.88 ± 0.76
*Trichoderma* sp. 25		13.97 ± 0.10	14.58 ± 0.88	3.72 ± 0.31	8832.63 ± 9.52	50.46 ± 0.01
*Aspergillus* sp. 26		0.02 ± 0.10	0 ± 0	4.88 ± 0.19	5855.12 ± 34.54	67.16 ± 0.01
*Aspergillus* sp. 27		0.02 ± 0.10	0 ± 0	4.63 ± 0.29	2058.28 ± 5.95	88.44 ± 0.01
*Aspergillus* sp. 28		1.48 ± 0.20	1.75 ± 0.11	4.68 ± 0.17	1458.78 ± 2.38	68.57 ± 1.50
*Aspergillus* sp. 29		1.48 ± 0.29	3.00 ± 1.19	0 ± 0	0 ± 0	82.92 ± 0.89
*Aspergillus* sp. 30		3.08 ± 0.29	6.03 ± 1.37	4.78 ± 0.19	159.87 ± 0.60	69.70 ± 1.13
*Penicillium* sp. 31		0.98 ± 0.09	0 ± 0	4.83 ± 0.20	3057.45 ± 32.44	82.52 ± 0.57
*Penicillium* sp. 32		0.10 ± 0.09	0 ± 0	4.88 ± 0.21	5855.12 ± 23.47	66.72 ± 0.63
*Penicillium* sp. 33		2.07 ± 0.08	3.25 ± 0.20	4.93 ± 0.22	1059.12 ± 20.94	73.69 ± 0.60
*Penicillium* sp. 34		1.48 ± 0.18	0 ± 0	4.98 ± 0.17	7453.78 ± 59.50	66.39 ± 2.16
*Penicillium* sp. 35		0.02 ± 0.08	0 ± 0	5.03 ± 0.16	4256.45 ± 19.05	63.37 ± 2.58
*Mucor* sp. 36		0.10 ± 0.38	0 ± 0	5.08 ± 0.15	1958.37 ± 59.50	66.35 ± 2.08
*Mucor* sp. 37		3.10 ± 0.58	1.82 ± 0.12	3.13 ± 0.14	5255.62 ± 59.50	63.00 ± 2.00
*Mucor* sp. 38		4.70 ± 0.55	6.07 ± 0.37	4.18 ± 0.33	3996.67 ± 39.99	62.34 ± 2.08
Tukey’s HSD test	*p*-value	0.00*	0.00*	0.00*	0.00*	0.00*
	MSD	0.03	0.29	0.44	5.78	3.63

*Significant differences (α ≤ 0.05, *n* = 38). The minimum significant difference (MSD) was utilized for pairwise comparisons, applying Tukey’s HSD test.

### 3.3 Identification of *Trichoderma* sp. WNZ-21

#### 3.3.1 Morphological and microscopic features

**FIGURE 2:**
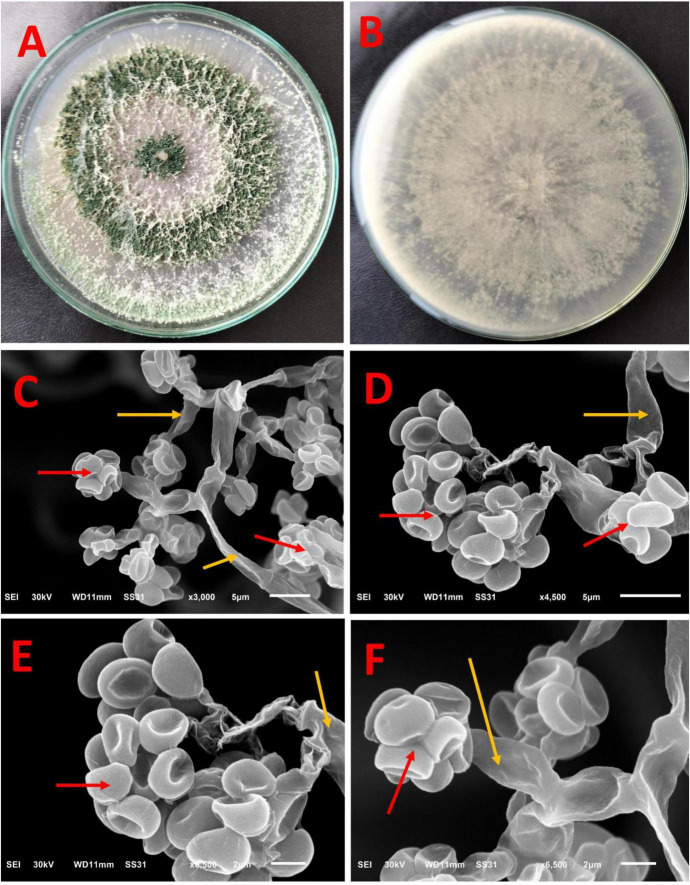
Macroscopic and ultrastructure of *Trichoderma* sp. WNZ-21 after 5 days of growth at 28 ± 2°C. Macroscopic view: **(A)** front view and **(B)** reverse view. Scanning electron microscopy under different magnifications **(C–F)** shows conidia (red arrows) and phialides (yellow arrows).

The macroscopic characteristics of the front and reverse views of *Trichoderma* sp. WNZ-21 (Figures 2A,B) shows the development of dense conidia, with a green center and white conidia towards the periphery. The reverse of the colony is creamy and often folded or convoluted. No pigment diffusion is observed on the PDA plate. There is neither aerial mycelium nor sweet coconut odor.

The SEM (Figures 2C–F) shows phialides, produced at the tips of these branches and arranged in whorls of 2–4, are straight, ampulliform, and slightly enlarged in the middle, measuring an average of 9–12 micrometers long and 1–3 micrometers wide. This isolate does not contain any intercalary phialides. Additionally, dark green conidia were observed. These conidia, globose to sub-globose or ovoidal in shape, measure between 3 and 3.5 micrometers long and 2–2.5 micrometers wide. The red arrows refer to conidia, while the yellow arrows refer to phialides.

#### 3.3.2 Molecular identification

**FIGURE 3:**
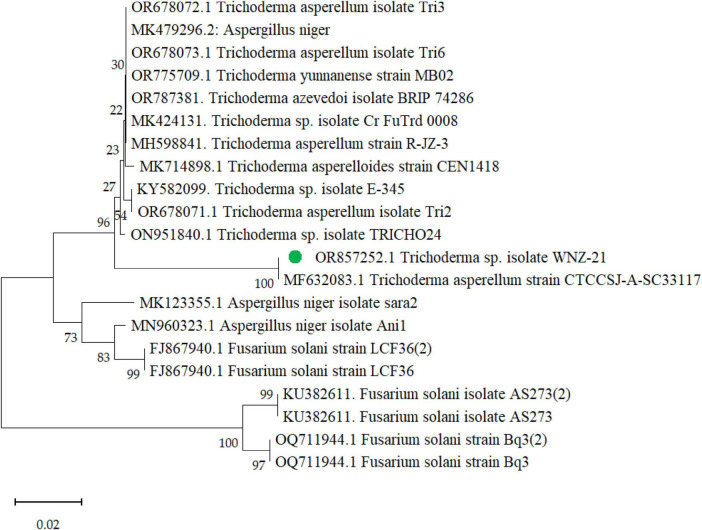
Phylogenetic tree of *Trichoderma asperellum* WNZ-21 (designated in green dot) and related strains based on ITS region sequences with accession number OR857252.

The BLAST analysis of the ITS region against the NCBI nucleotide database (Supplementary Table 1) revealed a high degree of similarity among the analyzed sequences with a consistently significant E-value of zero across all comparisons. Notably, eight out of the twelve most similar sequences corresponded to *T. asperellum*, while the remaining four belonged to the same genus (*Trichoderma* sp.) but lacked species-level identification. This data strongly supports the fungus as *T. asperellum* based on the ITS region. Further analysis identified *T. asperellum* strain MF632083.1 as the closest match, exhibiting a significantly higher sequence identity (97.88%) compared to other strains (94.42 to 94.62%). Importantly, all strains, including WNZ-21, displayed 100% query coverage, further solidifying the identification. The phylogenetic tree (Figure 3) depicts the relationship between *T. asperellum* WNZ and other related strains based on genetic markers. It is positioned closely to *T. asperellum* strain MF632083. This visual representation corroborates with BLAST analysis (Supplementary Table 1), highlighting a closer genetic affinity between these two strains compared to others. However, the phylogenetic analysis, together with ITS and BLAST, is consistent with the previous morphological characteristics, confirming the identification as *T. asperellum*.

**FIGURE 4:**
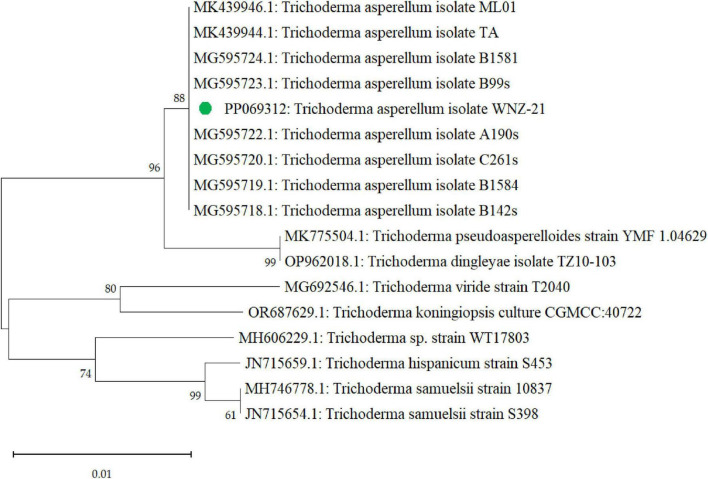
A phylogenetic tree that was generated through sequence analysis of the *tef1* gene illustrates the positioning of the *Trichoderma asperellum* isolate identified via the gene sequence (PP069312, green dot) among the related sequences.

Furthermore, the phylogenetic exploration based on the *tef1* gene confirmed the identification of our isolate (PP069312) as *T. asperellum*. BLAST analysis (Supplementary Table 2) showed high sequence similarity (99–100%) to other *T. asperellum* sequences in GenBank. Phylogenetic analysis (Figure 4) placed our isolate within a monophyletic *T. asperellum* clade with strong bootstrap support, indicating a close evolutionary relationship with other members of this species. Our isolate formed a distinct subclade within the *T. asperellum* group, suggesting potential genetic divergence and potentially unique characteristics warranting further investigation.

### 3.4 Analysis of *T. asperellum* filtrate

#### 3.4.1 Amino acid profile

**FIGURE 5:**
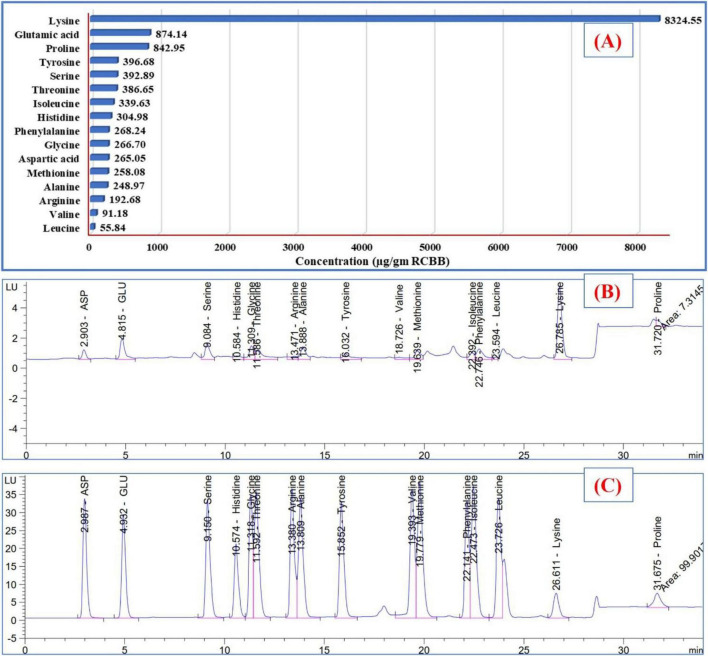
The amounts **(A)** of amino acids detected in the filtrate of the fungus grown on the residue of common bean biomass, and **(B)** HPLC chart, and amino acids standard chart **(C)**.

The degradation of RCBB by *T. asperellum* released 16 AAs (Figure 5), with a total amount being 13509.21 μg/g RCBB. Common AAs include glutamic acid, aspartic acid, serine, glycine, threonine, arginine, histidine, alanine, tyrosine, and methionine. Other notable AAs included cystine, valine, isoleucine, phenylalanine, leucine, lysine, and proline. Lysine was the most abundant individual compound, accounting for almost 30.76% of the total area. Eight of these AAs are essential for human health, while eight are non-essential. Four of the non-essential AAs are conditionally essential. The highest concentrations of AAs were found in lysine (8324.55 μg/g RCBB), glutamic acid (874.14 μg/g RCBB), proline (842.95 μg/g RCBB), tyrosine (396.68 μg/g RCBB), serine (392.89 μg/g RCBB), and threonine (386.65 μg/g RCBB).

#### 3.4.2 GC-MS analysis of the fungal filtrate

GC-MS confirmed RCBB degradation by *T. asperellum* WNZ-21, identifying 17 metabolites (Table 2, Supplementary Table 3, and Supplementary Figure 2). Key bioactive entities included D-Pinitol (47.04%) and erythro-pentonic acid derivatives (18.65%). The remaining components include a diverse array of trace fatty acids and ester derivatives. These findings highlight the fungus’ capacity to transform plant biomass into structurally diverse metabolites with therapeutic potential. However, Future studies will prioritize isolation and HR-MS/NMR characterization.

**TABLE 2:** GC-MS analysis of *Trichoderma asperellum* filtrate grown on the residue of common bean biomass.

Peak	RT	Name	Formula	Area	Area sum %
1	12.096	(2S,3R)-3-[(4E,7E)-Nona-4,7-dienoyl]-N,N-bis(trimethylsilyl)oxirane-2-carboxamide	C_18_H_33_NO_3_Si_2_	1953539.02	5.34
2	12.695	Silane, [(1-methoxy-1,3-propanediyl)bis(oxy)]bis[trimethyl-	C_10_H_26_O_3_Si_2_	1380409.33	3.77
3	16.619	Glycerol, 3TMS derivative	C_12_H_32_O_3_Si_3_	4308883.83	11.77
4	21.415	Erythro-Pentonic acid, 2-deoxy-3,4,5-tris-O-(trimethylsilyl)-, trimethylsilyl ester	C_17_H_42_O_5_Si_4_	6826323.09	18.65
5	22.113	Tartaric acid, 4TMS derivative	C_16_H_38_O_6_Si_4_	1523097.7	4.16
6	23.297	D-Glucitol, 6TMS	C_24_H_62_O_6_Si_6_	442117.87	1.21
7	23.54	Terephthalic acid, 2TMS derivative	C_14_H_22_O_4_Si_2_	300555.86	0.82
8	23.616	Azelaic acid, 2TMS derivative	C_15_H_32_O_4_Si_2_	191816.93	0.52
9	24.268	D-Pinitol, pentakis(trimethylsilyl) ether	C_22_H_54_O_6_Si_5_	17219216.94	47.04
10	24.837	Cyclopropanedodecanoic acid, 2- octyl-, methyl ester	C_24_H46O_2_	293633.03	0.8
11	25.277	D-Mannitol, 6TMS	C_24_H_62_O_6_Si_6_	181570.03	0.5
12	26.029	Palmitic Acid, TMS derivative	C_19_H_40_O_2_Si	400741.97	1.09
13	26.507	9-Octadecenoic acid (Z)-, 2-hydroxy-1-(hydroxymethyl)ethyl ester	C_21_H_40_O_4_	248368.11	0.68
14	27.804	Stearic acid, TMS derivative	C_21_H_44_O_2_Si	482820.13	1.32
15	30.961	Behenic acid, TMS derivative	C_25_H_52_O_2_Si	176556.12	0.48
16	32.38	Lignoceric acid, TMS derivative	C_27_H_56_O_2_Si	199609.91	0.55
17	36.152	Silane, diethylheptyloxyoctadecyloxy-	C_29_H_62_O_2_Si	473527.61	1.29

#### 3.4.3 Phytochemical and FT-IR spectral analysis

Finally, the fungal filtrate exhibited significant phytochemical content (Supplementary Figure 3), including phenolics (55.22 mg/g), flavonoids (11.22 mg/g), and tannins (18.90 mg/g). FT-IR analysis (Supplementary Figure 4 and Supplementary Table 4) corroborated these findings, revealing NH/C-N stretches (3362–1231 cm^−1^) indicative of amino acids and proteins, C = O bands (1,713–1,645 cm^–1^) from carboxylic acids and peptides, and polysaccharide-associated C-O stretches (1,066 cm^–1^). These results align with GC-MS-detected metabolites (e.g., D-Pinitol, fatty acids) and underscore the filtrate’s chemical complexity.

### 3.5 Biological activity of the fungal filtrate

#### 3.5.1 Cytotoxic study on cell lines

The MTT assay was applied, and doxorubicin was utilized as a standard drug (Supplementary Figure 5). As a general rule, there was a negative correlation (r) between cell line viability and the concentration of the fungal filtrate. The values of r were −0.9380, −0.8967, and −0.8567 (*p* ≤ 0.05) for WI38, HePG2, and MCF7, respectively.

The average of relative viability reached 47.5 ± 1.6, 29.8 ± 1.2, and 23.4 ± 0.4 at 100 μg/mL of fungal filtrate for WI38, HePG2, and MCF7 cell lines, respectively. Lower concentrations resulted in higher cell viability. No cytotoxicity was recorded at 6.25 (WI38), 3.125 (HePG2), and 1.56 (MCF7) μg/mL.

**FIGURE 6:**
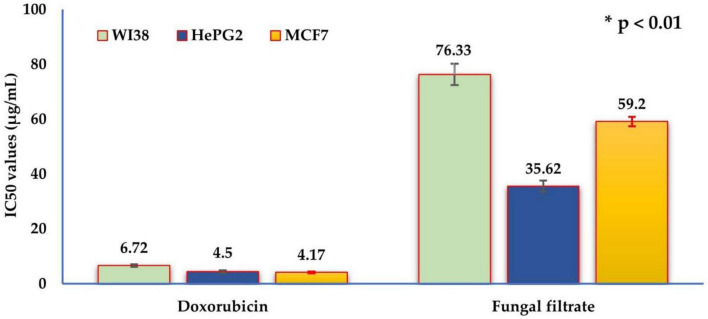
The IC_50_ values of the *in vitro* cytotoxic activity of *Trichoderma asperellum* filtrate and standard doxorubicin drug against the three human cell lines: human lung fibroblast (WI38), hepatocellular carcinoma (HePG2), and mammary gland breast cancer (MCF7). The half maximal inhibitory concentration (μg/mL) categories are very strong (1–10), strong (11–20), moderate (21–50), weak (51–100), and non-cytotoxic (>100).

Furthermore, the cytotoxic potency of the fungal filtrate against the three cell lines was calculated (Figure 6), where the IC_50_ values were 76.33 ± 3.9, 35.62 ± 2.1, and 59.20 ± 1.7 μg/mL for WI38 (normal cell line), HePG2, and MCF7 (tumor cell lines), respectively. Notably, the fungal filtrate was weak against the normal cell line, whereas it exhibited selective cytotoxicity against tumor cell lines.

#### 3.5.2 Anti-hemolytic activity

The results of the erythrocyte hemolysis activity of blood samples (Table 3) indicated a relatively low hemolysis percentage of fungal filtrate (4.40 ± 0.69%), indicating reasonably anti-hemolytic activity. However, L-ascorbic acid showed lower hemolysis as an antioxidant and can protect cells, including red blood cells, from oxidative damage.

**TABLE 3:** Erythrocyte hemolysis activity of *Trichoderma asperellum* fungal filtrate.

Compound	Erythrocyte hemolysis		Anti-hemolysis, %
	Absorbance at 540 nm	Hemolysis, %	
Distilled water control	0.875	−	
L-ascorbic acid	0.034	3.88 ± 0.46	96.12
Fungal filtrate	0.126	4.40 ± 0.69	95.60

The hemolytic percentage in relation to distilled water was included as maximal hemolytic control (*n* = 6).

#### 3.5.3 Anti-inflammatory activity on rats

**FIGURE 7:**
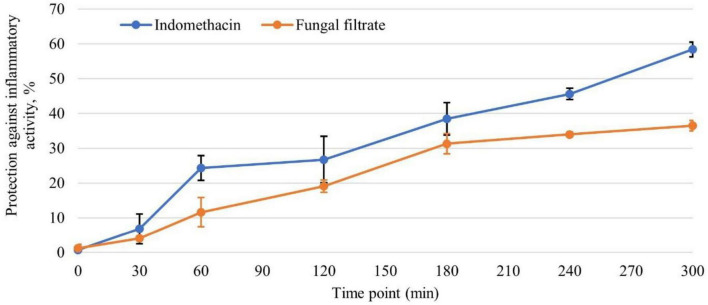
The efficacy of *Trichoderma asperellum* filtrate in protecting against inflammatory activity at different time points, in comparison with the standard indomethacin drug (*n* = 6).

The carrageenan-induced paw edema assay on rats (Figure 7) demonstrated a time-dependent anti-inflammatory response elicited by both indomethacin and the tested fungal filtrate. At zero time, a baseline level of protection is evident in both groups, with the fungal filtrate showing marginally higher protection (indomethacin: 0.71%, fungal filtrate: 1.18%). As the experiment progresses, both substances exhibit an increase in protection percentages. Notably, indomethacin demonstrates a consistent and pronounced rise in protection, reaching 58.41 % after 5 h. *T. asperellum* WNZ-21 filtrate also exhibits anti-inflammatory action, although at a lower level of protection compared to indomethacin. At the 5-h point, the fungal filtrate demonstrates a protection percentage of 36.50%.

## 4 Discussion

Endophytes, nonharmful microbes residing within plant tissues throughout part or all of their life cycle, represent a significant and often underexplored source of diverse enzymes and secondary metabolites with unique features and therapeutic potential (Al-Askar et al., 2022; Alrdahe et al., 2024; Moussa et al., 2023). Recognizing this vast potential and the pressing need for sustainable strategies for agricultural residue valorization, the central aim of this study was to explore a novel approach: the bioconversion of RCBB into valuable bioactive compounds. We specifically investigated the potential of using SSSF mediated by a selected potent endophytic fungus to achieve this goal and identify multi-bioactive metabolites.

Initially, the chemical composition of RCBB used in the current study aligned with previous studies (de Castro et al., 2016; de castro et al., 2021; Ferro et al., 2020; Santos et al., 2018) with slight differences. These differences may be attributed to the variety, growing season, and the ratio of plant parts.

Our initial investigation into the endophytic fungal community of common bean revealed the predominance of *Trichoderma* spp., consistent with their known metabolic versatility and prevalence in plant hosts, further supporting our focus on this genus. In comparison, 26 fungal species were recovered from the peanut plants, and some of the isolates produced active metabolites, including enzymes and citric acid (Al-Askar et al., 2022).

All isolates grew well and produced protease and released AAs, which are the key enzymes/factors in the biomass breakdown (El-Hersh et al., 2016; El-Hersh et al., 2014; Negm et al., 2021). The ability of the endophytic fungi to degrade RCBB using SSSF was confirmed. The SSSF technique is rarely used, especially with endophytes. It was chosen here due to its simplicity, low energy needs, and ability to mimic the natural growth environment of the fungi (AbdAl-Aziz et al., 2012; Negm et al., 2021).

Compared to the other isolates, which showed moderate degradation, the exceptional biomass degradation efficiency of isolate WNZ-21 underscores its role in biomass breakdown and the synthesis of secondary metabolites.

*Trichoderma* sp. WNZ-21 had an efficient enzyme profile and degraded RCBB, thus selected for further studies. RCBB is a complex substrate rich in hemicellulose, cellulose, and protein, its degradation requires several hydrolytic enzymes (cellulase, xylanase, and protease). While enzyme activity varied among isolates, most produced the three enzymes. The significant variation observed in the hydrolytic profile among different fungi may be attributed to differences in strain types and genetic variations (Al-Askar et al., 2022). Cellulose is broken down by cellulases into individual glucose monomers (Al-Askar et al., 2022). Likewise, xylanase facilitates the breakdown of hemicellulose (xylan), releasing xylose units (Abdelwahed et al., 2011; Bailey et al., 1992). Proteases break down the protein component of plant tissues into free AAs and peptides (Al-Askar et al., 2021) using endopeptidases and exopeptidases. Exopeptidases catalyze the terminal peptide bonds, while endopeptidases catalyze the non-terminal peptide bonds (El-Hersh et al., 2014). The synergistic action of these hydrolytic enzymes resulted in the breakdown of plant tissues into free AAs (Al-Askar et al., 2021; Al-Askar et al., 2022; Moussa et al., 2021).

*Trichoderma* sp. WNZ-21 was identified as *T. asperellum* based on morphological, cultural, and SEM examination. While these methods provide valuable insights, definitive fungal identification typically relies on a combination of morphological analysis and molecular techniques. The ITS region is a well-established fungal barcode due to its high sequence variability and conserved flanking regions, enabling accurate and reliable species-level identification (AbdAl-Aziz et al., 2012; Raja et al., 2017; Saber et al., 2010; Tretter et al., 2012).

Molecular identification *via* ITS (GenBank: OR857252) and *tef1* gene (GenBank: PP069312) sequencing confirmed the isolate as *T. asperellum* WNZ-21. Phylogenetic analysis resolved species-level ambiguities, aligning with prior taxonomic frameworks (De Bruyn et al., 2014; Hermosa et al., 2004). ITS is a valuable molecular marker for identifying *Trichoderma* species, highlighting the resolution and accuracy at the species level, while *tef1* is often employed in multiplex PCR assays for the precise differentiation of *Trichoderma* species (Hermosa et al., 2004; Letunic and Bork, 2021).

The fungal filtrate was analyzed for bioactive metabolites. The presence of these compounds indicated the successful degradation of plant biomass during fermentation.

The phytochemical content of the filtrate displayed levels of phenolics comparable to or exceeding values reported for *T. harzianum* filtrates (Lakhdari et al., 2023) and surpasses those of *T. harzianum* and *Glomus versiforme* (Omomowo et al., 2020), suggesting the antioxidant potential of phenolic compounds (Lakhdari et al., 2023). Flavonoid content aligns with the level documented for *T. harzianum* filtrates (Lakhdari et al., 2023). Flavonoids have health-promoting properties with antioxidant capacity (Elattar et al., 2024; Lakhdari et al., 2023). Tannin of the present isolate is considerably higher than those reported for *T. harzianum* filtrates and the *Calotropis procera* filtrate, where tannins can have diverse effects depending on their type and interaction with other components (Nofal et al., 2023). The fungal filtrate under study demonstrates a promising phytochemical profile, suggesting potential for further exploration.

The release of AAs and biomass reduction (%) provides further evidence of the degradation during fungal fermentation. However, the type and the amounts of AAs vary depending on the substrate, microbe, and fermentation settings. For instance, 14 AAs were released during the fermentation of chicken feathers by *Bacillus paramycoides*, where proline and aspartic acid had the highest concentrations of the released AAs (Moussa et al., 2021). Thrane et al. (2001) identified aspartic acid, glutamic acid, threonine, and alanine. However, the variation of the AAs profile suggests potential strain-specific variation in metabolic pathways. Furthermore, several AAs are reported to have antifungal activity, suggesting potential biocontrol capabilities (Moo-Koh et al., 2022).

While native RCBB contains 15.80% crude protein and 37.50% crude fiber, its recalcitrant lignocellulosic matrix prevents the spontaneous release of bioactives, as confirmed by the non-inoculated control. Fungal degradation via robust hydrolytic unlocks structural components, generating monomers that fuel secondary metabolism. While this study does not present time-course enzyme activity data, the 10-day SSSF protocol aligns with established fungal bioprocess timelines, where peak hydrolytic activity typically occurs within 3–5 days (Alrdahe et al., 2024; El-Naggar et al., 2014; Saber et al., 2015). The biomass degradation by *T. asperellum* WNZ-21 (67.19% RCBB), for instance, positively correlates with its enzymatic activity and TFAA yield. For instance, AAs and phenolic liberation are direct consequences of protease- and ligninolytic-driven degradation, respectively, which concluded that fungal enzyme activity and substrate degradation are interdependent processes (De Silva et al., 2019; Moussa et al., 2023). Future studies will incorporate kinetic enzyme assays and substrate degradation profiles to optimize bioprocessing parameters, building on the current findings.

The genus *Trichoderma* exhibits a diverse metabolite profile under standard growth conditions (25°C, pH 6.0, glucose-based media), characterized predominantly by peptaibols, polyketides, terpenes, and volatile organic compounds (Moussa et al., 2023; Reino et al., 2008). Key metabolites include 6-pentyl-α-pyrone, harzianic acid, harzianolide, and various trichothecenes that contribute to their biocontrol capabilities (Vinale et al., 2014). Peptaibols such as alamethicin and trichokonin demonstrate significant antimicrobial activity through membrane permeabilization (Zhishen et al., 1999). The secondary metabolome also features gliotoxin, gliovirin, and koninginins, which vary in concentration depending on species and strain (Zeilinger et al., 2016). Environmental factors substantially influence metabolite production patterns, with carbon source availability and nitrogen limitations particularly affecting the biosynthesis of antifungal compounds (Mukherjee et al., 2013). Recent metabolomic analyses have revealed over 300 unique compounds in *T. harzianum* alone, highlighting the chemical complexity underlying Trichoderma’s ecological success as both a saprophyte and mycoparasite (Contreras-Cornejo et al., 2016).

However, our GC-MS metabolite profile *of T. asperellum* WNZ-21 during RCBB fermentation diverges significantly from this baseline. This difference might be attributable to the variation in the growth media and the fungal origin. Endophytes surpass other fungi by generating a unique range of secondary metabolites (peptaibols, gliotoxin, gliovirin, polyketides, pyrones, and terpenes) with varied bioactivities on minimal nutrients, offering diverse applications, e.g., pharmaceuticals, and plant growth regulation (De Silva et al., 2019; Sridharan et al., 2021; Vinale et al., 2008; Vizcaino et al., 2005). The GC-MS analysis indicated that only four components were reported in previous studies. Palmitic acid TMS derivative is present in brown seaweeds and the associated endophytic fungi (Teixeira et al., 2019). D-Pinitol, pentakis (trimethylsilyl), the major component of the fermented filtrate, is present in carob syrups (Christou et al., 2019). D-Mannitol, 6TMS was reported in olive leaf extract (Islamčević Razboršek et al., 2015). Silane, diethylheptyloxyoctadecyloxy- was reported in the leaves extract of *Murraya koenigii* (Prabaharan et al., 2023). The last three compounds identified in this filtrate are, to our knowledge, unreported in other microbial filtrates, while the remaining 13 are neither discovered in microbes nor plants. This may be due to the unique features of the endophytic fungus and suggesting a wide range of potential bioactivities. While structural confirmation via NMR/HR-MS is warranted, their bioactivities (anticancer, anti-hemolytic) provide functional evidence for prioritizing follow-up research. This aligns with the study’s objective to establish a bioprocess framework for valorizing agricultural waste, with structural studies reserved for later phases.

FT-IR signatures of the *T*. *harzianum* filtrate revealed the presence of various aliphatic chains and amino functional groups characteristic of AAs. Recent literature indicated the presence of AAs and other nitrogenous compounds by *Trichoderma* species, aligning with our observations of NH, CH, C = O, and C-N stretches in the FT-IR spectrum, suggesting the production of metabolites and secondary metabolites, which could contribute to the diverse peaks beyond those indicative of amino acids in the FT-IR spectrum (Abdel-Kareem and Zohri, 2018; Alrdahe et al., 2024; Elattar et al., 2024; Konappa et al., 2021; Tomah et al., 2020). Furthermore, FT-IR signatures (e.g., N-H bends, C = C stretches) suggest potential contributions from alkaloids, terpenoids, and peptides. FT-IR analysis of the *T*. *harzianum* culture filtrate revealed the presence of polysaccharides (Saravanakumar et al., 2021). Another study observed notable changes in the total protein of *Trichoderma* spp.-treated tomato seed radicles, accompanied by a parallel reduction in pectin and/or xyloglucan levels (Vukelić et al., 2024).

This study utilized the SSSF system integrating *T. asperellum* WNZ-21 with RCBB and mineral salts. This system was specifically designed to mimic natural fungal degradation processes and prioritize the synergistic interplay between fungal enzymes, such as cellulase, xylanase, and protease, and substrate breakdown, rather than isolating individual contributors. Crucially, non-inoculated controls confirmed that mineral salts and unfermented RCBB exhibited no hydrolytic activity or bioactivity, thereby validating fungal metabolism as the direct driver for the observed effects and the accumulation of diverse metabolites, including amino acids and a broad spectrum of secondary metabolites. Furthermore, the compounds identified via GC-MS were unambiguously determined to be of fungal origin, distinguishing them from any pre-existing plant-derived phytochemicals like phenolics or flavonoids. Beyond elucidating the fungal degradation pathways within this SSSF framework, this research comprehensively examines the biological properties of the resultant filtrate, whose bioactivity suggests potential therapeutic and health benefits derivable from these transformed plant residues. Consequently, the focus on the holistic fungal-plant biomass interaction and the characterization of its multi-bioactive products not only underscores the novelty and broader implications of valorizing agricultural waste but also highlights the complex and multifaceted nature of natural bioactivity for sustainable biotechnology and diverse applications.

The filtrate exhibited selective cytotoxicity against tumor cell lines (HePG2 and MCF7) than normal cells (WI38). AAs play a complex role in cancer, impacting energy production, redox balance, epigenetic regulation, and immune responses (Lieu et al., 2020). While AA restriction shows promise in some contexts (Guillén-Mancina et al., 2023), AAs also support healthy vasculature (Durante, 2020) and can contribute to treatment resistance (Yoo and Han, 2022). Notably, specific AAs like lysine (Abd El-Aal et al., 2023), glutamic acid (Dutta et al., 2013), and proline (Yin et al., 2022) exhibit anti-cancer potential, highlighting the need for further research into manipulating AA metabolism for effective cancer treatment. Furthermore, some GC-MS-detected components, such as stearic acid, a saturated fatty acid, have been reported to hinder the growth of human breast cancer cells *in vitro* (Khan et al., 2013), whereas palmitic acid has anticancer activity against prostate and breast cancer (Zafaryab et al., 2019; Zhu et al., 2021).

In the realm of anticancer activities, both AAs and secondary metabolites play crucial roles through diverse mechanisms. Lysine not only exhibits antibacterial effects but also contributes to anticancer effects by modulating AAs metabolism within cancer cells, effectively starving tumors of essential nutrients (Yoo and Han, 2022). Glutamic acid also demonstrates anticancer potential by interfering with glutamine metabolism, a pathway vital for cancer cell proliferation (Dutta et al., 2013). Building upon these mechanisms, phenolic compounds exhibit anticancer effects through multiple pathways, including reactive oxygen species (ROS) generation, DNA damage induction, and apoptosis activation in tumor cells (Lakhdari et al., 2023; Lieu et al., 2020; Yin et al., 2022). Flavonoids contribute to anticancer activity by inducing cell cycle arrest at the G2/M phase and inhibiting angiogenesis via downregulation of vascular endothelial growth factor signaling (Lakhdari et al., 2023; Saravanakumar et al., 2021). Tannins demonstrate anticancer potential through suppression of the NF-κB signaling pathway, which reduces tumor-associated inflammation and metastatic potential (Ekambaram et al., 2022). Similarly, tyrosine derivatives, particularly tyrosine kinase inhibitors, exert anticancer effects by blocking critical oncogenic pathways (Yin et al., 2022; Yoo and Han, 2022). Finally, methionine inhibits cancer cell proliferation by altering redox homeostasis through glutathione depletion and oxidative stress amplification (Durante, 2020; Wu et al., 2003).

Antioxidant properties are another significant feature, particularly of some AAs. Proline acts as a direct antioxidant by scavenging free radicals, thereby protecting cells from oxidative damage and indirectly supporting anticancer effects by reducing oxidative stress-driven mutations (Yin et al., 2022). Tyrosine, with its phenolic hydroxyl group, also neutralizes ROS and contributes to antioxidant defense (Cui et al., 2019). Methionine, with its sulfur-containing structure, regenerates glutathione, a key cellular antioxidant, further solidifying its role in combating oxidative stress (Wu et al., 2003). The provided information on secondary metabolites emphasizes their antioxidant properties, such as ROS, suggesting an indirect modulation of the cellular redox balance (Cui et al., 2019; Wu et al., 2003; Yin et al., 2022).

Various other molecules also exhibit significant anticancer activities. Fatty acid derivatives and related compounds, cyclopropanedodecanoic acid, 2- octyl-, methyl ester, have shown the potential to induce apoptosis in cancer cells (Zafaryab et al., 2019). Both palmitic acid, TMS derivative, and Stearic acid, TMS derivative, demonstrate anticancer effects, with palmitic acid inhibiting cancer cell proliferation and stearic acid derivatives inhibiting tumor growth (Zafaryab et al., 2019). These diverse compounds, working through various mechanisms, highlight the complexity of anticancer strategies derived from natural sources.

The GC-MS-detected compounds also showed antioxidant properties, contributing to cellular defense against oxidative stress. (2S,3R)-3-[(4E,7E)-Nona-4,7-dienoyl]-N,N-bis(trimethylsilyl)oxirane-2-carboxamide, D-Pinitol, pentakis(trimethylsilyl) ether, and D-Mannitol, 6TMS all exhibit radical scavenging activity (Copini et al., 2020; Ramos-Escudero et al., 2021). Glycerol, TMS derivative, acts as a hydrogen donor to neutralize ROS (Ramos-Escudero et al., 2021), while tartaric acid, TMS derivative, scavenges ROS and enhances cellular defense mechanisms (Saffari and Saffari, 2020). Erythro-Pentonic acid, 2-deoxy-3,4,5-tris-O-(trimethylsilyl)-, trimethylsilyl ester chelates metal ions to reduce oxidative stress, and D-Glucitol, TMS stabilizes free radicals (Ramos-Escudero et al., 2021). This diverse array of compounds underscores the multifaceted nature of antioxidant defenses.

However, while the studied fungal filtrate exhibits promisingly lower toxicity against normal cells, its cytotoxic mechanism against WI38, HePG2, and MCF7 cells remains unclear. Further, *in vivo* studies are warranted to investigate potential allergenic and immunomodulatory effects.

Anti-hemolytic activity, another biological assay, evaluates a material’s ability to protect RBCs from lysis. Hemolysis, if uncontrolled, can lead to complications such as anemia, jaundice, gallstones, and, in severe cases, organ failure (Fattizzo and Barcellini, 2022; Weisel and Litvinov, 2019). Mechanistically, anti-hemolytic activity may arise from free radical scavenging, oxidative stress reduction, RBC membrane stabilization, and inhibition of hemolytic agents (Karim et al., 2020; Yang et al., 2005). The observed anti-hemolytic activity of the *T. asperellum* WNZ-21 filtrate (4.40 ± 0.69% hemolysis) is attributed, at least in part, to glutamic acid derivatives and lysine, both of which exhibit protective effects (Neubauer et al., 2020). Specifically, glutamic acid derivatives (Elsayed et al., 2023) prevent RBC breakdown, while lysine (Wan et al., 2022) inhibits RBC lysis by stabilizing cell membranes. These findings align with the current filtrate’s rich amino acid profile, underscoring its potential to mitigate oxidative damage and hemolytic pathology.

Anti-inflammatory activities represent another important facet of bioactivity. Several of the mentioned compounds demonstrate anti-inflammatory effects through various mechanisms. (2S,3R)-3-[(4E,7E)-Nona-4,7-dienoyl]-N,N-bis(trimethylsilyl)oxirane-2-carboxamide, Azelaic acid, TMS derivative, and D-Pinitol, pentakis(trimethylsilyl) ether may modulate cytokine pathways or inhibit pro-inflammatory mediators (Manivannan et al., 2017). 9-Octadecenoic acid (Z)-, 2-hydroxy-1-(hydroxymethyl)ethyl ester suppresses pro-inflammatory cytokines (Manivannan et al., 2017), while Stearic acid, TMS derivative reduces inflammation via lipid mediator regulation (Pan et al., 2010). These diverse compounds offer various avenues for mitigating inflammatory responses.

The anti-inflammatory activity observed in the *T. asperellum* WNZ-21 filtrate may be attributed to its biomolecules, including lysine and glutamic acid, as well as palmitic acid. AAs are known to effectively neutralize pro-inflammatory bacterial components like lipopolysaccharides and lipoteichoic acid. This neutralization likely contributes to the observed anti-inflammatory effect by downregulating the expression of key mediators of inflammation, such as cytokines, enzymes, and transcription factors (Brunetti et al., 2020; Das and Gavel, 2020; Wan et al., 2022; Yang et al., 2020; Younis and Saleh, 2021). Some fatty acids, such as stearic acid, have demonstrated anti-inflammatory action that could potentially mitigate the liver damage caused by inflammation associated with cholestasis (Pan et al., 2010). 9-Octadecenoic acid (Z)- derivatives exhibited anti-inflammatory properties in lipopolysaccharide-activated macrophages (Manivannan et al., 2017). This anti-inflammatory activity might be linked to modulation of the immune cell function and scavenging of reactive oxygen species (Yoshitomi and Nagasaki, 2014). While *T. asperellum* WNZ-21 filtrate exhibits anti-inflammatory activity, albeit less potent than indomethacin, its discernible protective effect warrants further investigation into underlying mechanisms and optimized dosage regimens for the potential development of novel anti-inflammatory treatments.

The fungal filtrate was found to be rich in enzymes. The therapeutic potential of proteases as anti-inflammatory agents is well-established, with specific variants being utilized as adjunctive treatments or supplements in clinical practice. While proteases have demonstrated anticancer properties in experimental models through mechanisms such as extracellular matrix modulation (Chobotova et al., 2010), they have not yet achieved widespread clinical application as primary anticancer therapeutics compared to specialized enzymes targeting metabolic pathways like L-asparaginase (Naser et al., 2020; Othman et al., 2022). Cellulases and xylanases, which hydrolyze structural cellulose and xylan specific to plant cell walls, have limited direct therapeutic relevance as anticancer, anti-inflammatory, or anti-hemolytic drugs due to the absence of their specific substrates within animal tissues.

Given the promising anti-hemolytic and anti-inflammatory activities demonstrated by the fungal filtrate, particularly considering that these properties have not, to our knowledge, been previously reported for fermented plant biomass residues, further investigation is warranted to elucidate the underlying mechanisms and fully assess the therapeutic potential.

Future research must prioritize *in vivo* toxicity, safety, and clinical trial assessments to confirm therapeutic potential. Cytotoxicity assessments, although showing selectivity, require expansion to a broader range of cell lines to fully understand mechanisms and refine applications. Furthermore, *in vivo* studies should address potential allergenic and immunomodulatory effects. The SSSF method, chosen for its advantages, presents scalability challenges concerning heat and mass transfer in industrial-scale vessels, necessitating optimization for process efficiency. Chemical characterization, while identifying 17 compounds, demands further structural validation and multi-biological activity analysis of these entities. Future research should therefore focus on: (1) purification and structural identification of active, (2) mechanistic elucidation of bioactivities; (3) SSSF process optimization and scale-up; and (4) investigation of host and environmental factors influencing metabolite production to enhance broader applicability.

## 5 Conclusion

This study addressed the critical need for bioactive compounds with multi-biological activities by exploring the potential of endophytic fungi to valorize proteinaceous agricultural waste, specifically the protein-rich RCBB. We investigated the ability of *T. asperellum* WNZ-21 to bioconvert RCBB through SSSF, achieving 67.19% biomass degradation, and profiled the multi-biological activity of the resulting filtrate.

Our findings demonstrate that *T. asperellum* WNZ-21, identified morphologically and molecularly (GenBank ITS: OR857252; *tef1* gene: PP069312), efficiently degraded RCBB via SSSF, yielding a metabolite-rich filtrate. This filtrate contained a significant amount of amino acids (total: 13,509.21 μg/g RCBB), notably lysine (8,324.55 μg/g RCBB), alongside high levels of phenolics (55.22 mg/g RCBB), flavonoids (11.22 mg/g RCBB), and tannins (18.90 mg/g RCBB). Importantly, GC-MS analysis revealed 17 compounds, highlighting the potential for discovering new chemical structures.

Biologically, the filtrate exhibited promising selective anticancer activity against HePG2 (IC_50_: 35.62 ± 2.1 μg/mL) and MCF7 (IC_50_: 59.20 ± 1.7 μg/mL) cells, showing reduced toxicity toward normal WI38 cells (IC_50_: 76.33 ± 3.9 μg/mL). It also demonstrated significant anti-hemolytic effects, resulting in only 4.40 ± 0.69% hemolysis, and notable anti-inflammatory effects (36.50% protection in a carrageenan-induced paw edema model). These observed activities are consistent with the presence of identified amino acids and secondary metabolites known for membrane stabilization and oxidative stress modulation.

This work successfully demonstrates the biotechnological potential of using endophytic fungi to convert agricultural waste into multi-bioactive compounds, advancing sustainable biotechnology and drug discovery efforts. This study provides a strong foundation, and future research will be crucial to isolate the reported compounds and confirm their structure, in-depth elucidate their specific mechanisms of action, optimize the fermentation process for scalability, and conduct comprehensive safety and efficacy studies for potential therapeutic applications.

## Data Availability

The datasets presented in this study can be found in online repositories. The names of the repository/repositories and accession number(s) can be found in the article/Supplementary material.

## Footnotes

1 National Center for Biotechnology Information. BLAST^®^ (Basic Local Alignment Search Tool). Retrieved Jan 3, 2025 from http://www.ncbi.nlm.nih.gov/BLAST

2 The ARRIVE (Animal Research: Reporting of In Vivo Experiments) guidelines. Retrieved Jan 3, 2025 from https://arriveguidelines.org
