# Supplementary material for: Endophytic Trichoderma asperellum WNZ-21 produces novel bioactives with anticancer, anti-inflammatory, and hemoprotective properties from fermented bean biomass
Source: Front Microbiol. 2025 Jun 10;16:1609361. doi: 10.3389/fmicb.2025.1609361 (PMC12185484; doi:10.3389/fmicb.2025.1609361)
Supplement: Supplementary file 1 [file Data_Sheet_1.docx]

Supplementary Material

**2. Results and discussion**

**Initial Fungal characterization**

***Trichoderma* spp.**

The general morphological characteristics of *Trichoderma* spp. were confirmed with the aid of (Asis et al., 2021; Siddiquee & Siddiquee, 2017). The macroscopic characteristics of the colony of *Trichoderma* species showed the development of scattered blue-green or yellow-green pigments (when growing on PDA plates), which can sometimes form concentric rings. Some species do not develop any pigment on the reverse side of the plate, while others develop reddish-brown pigment. The microscopic characteristics of the colony are typically circular, but it can be irregular or lobed in some species. The colony size is usually small to medium, but it can range from 2 to 10 centimeters in diameter, depending on the growth rate and the growth medium. The colony texture is typically flat, velvety, or powdery, but it can be cottony, woolly, or granular in some species. A conidiophore is a specialized hypha that bears conidia. It can be simple, branched, or clustered. It can have one or more phialides at the tip or along the sides.

***Aspergillus* spp.**

*Aspergillus* spp. showed the various features of Aspergilli depending on the species (Diba et al., 2007; Domsch et al., 1980; Samson & Pitt, 2000). The macroscopic appearance of Aspergillus colonies varies depending on the species. On PDA, they were flat, woolly, or powdery, and their color can range from yellow-green to blue-green, black, or cinnamon-brown. *Aspergillus* species have septate hyphae that branch at 45-degree angles. They produce conidiophores that bear vesicles at the tips. The vesicles produce phialides, which in turn produce conidia. The shape and size of the vesicles, phialides, and conidia vary among species and can be used to identify them.

***Penicillium* spp.**

The genus *Penicillium* spp. was identified compared with the previous work (de Hoog et al., 2005; Samson et al., 2014; St-Germain & Summerbell, 2003; Sutton et al., 1998). The macroscopic characteristics of *Penicillium* colonies are typically fast-growing, flat, filamentous, and have a velvety, woolly, or cottony texture. The color of the colonies varies depending on the species and can be dark green, greenish orange, olive-gray, or pinkish. The reverse side of the colonies is usually pale to yellowish. Regarding the microscopic features, the vegetative part of the fungus consists of a profuse branching of septate, cylindrical, and thin-walled hyphae. A long, filamentous-like structure that bears conidia. Asexual spores (conidia) are produced in chains on the flask-like phialides. A characteristic feature of *Penicillium* is the dense cluster of conidiophores that produce conidia. The youngest spores are at the base of the chain.

***Mucor* spp.**

Morphological characteristics of the genus *Mucor* spp. were identified according to (de Hoog et al., 2005; Sutton et al., 1998). Concerning the macroscopic features, *Mucor* colonies grow rapidly at 25-30°C and can quickly cover the surface of the agar. They have a fluffy appearance and can grow to be several centimeters tall, resembling cotton candy. The color of the colonies is initially white, but it can become grayish brown over time. The reverse side of the colonies is white. The microscopic features of Mucor species showed non-septate or sparsely septate hyphae that are 6-15 µm wide. They may also produce intercalary or terminal arthrospores (oidia), which are asexual spores that are formed by fragmentation of hyphae. Some species may also produce chlamydospores, which are thick-walled asexual spores that help the fungus survive adverse conditions. Mucor species do not have apophyses, rhizoids, or stolons. Sporangiophores are short, erect, and taper towards their apices. They may form short sympodial branches. Columellae are hyaline or dematiaceous, and they are not always visible. Mucor species can be differentiated by the branching pattern of their sporangiophores. Some species have unbranched sporangiophores, while others have branched sporangiophores.

**Screening the Hydrolytic Activity**


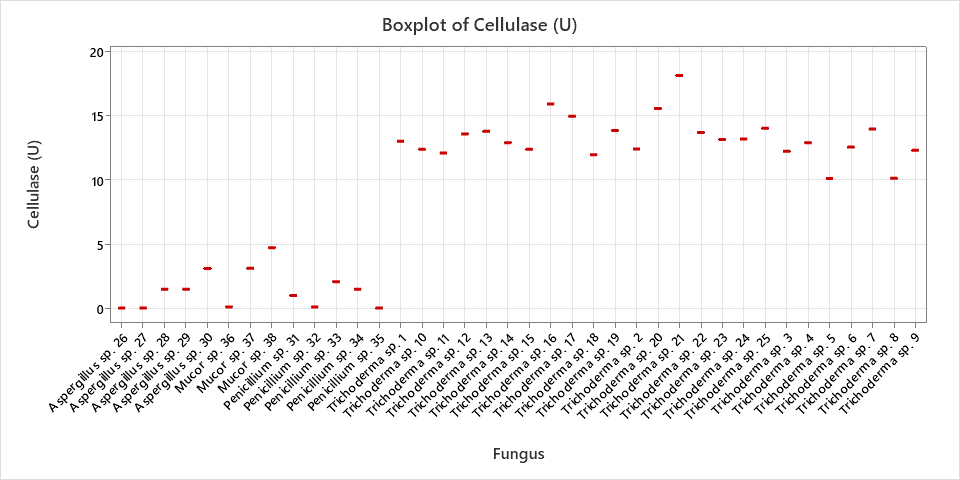


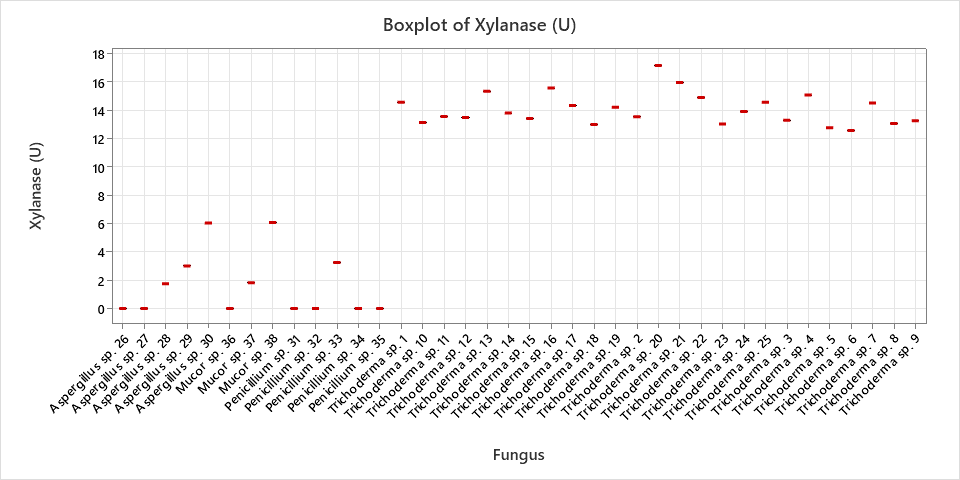


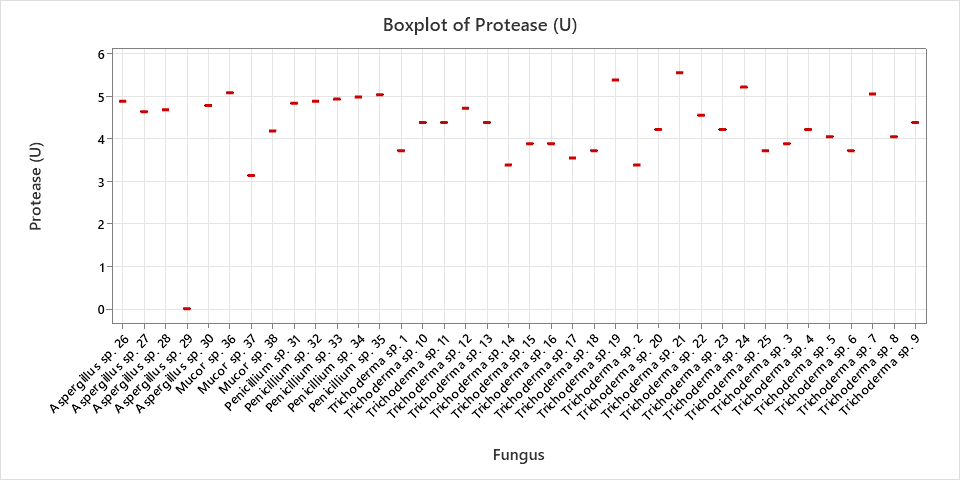


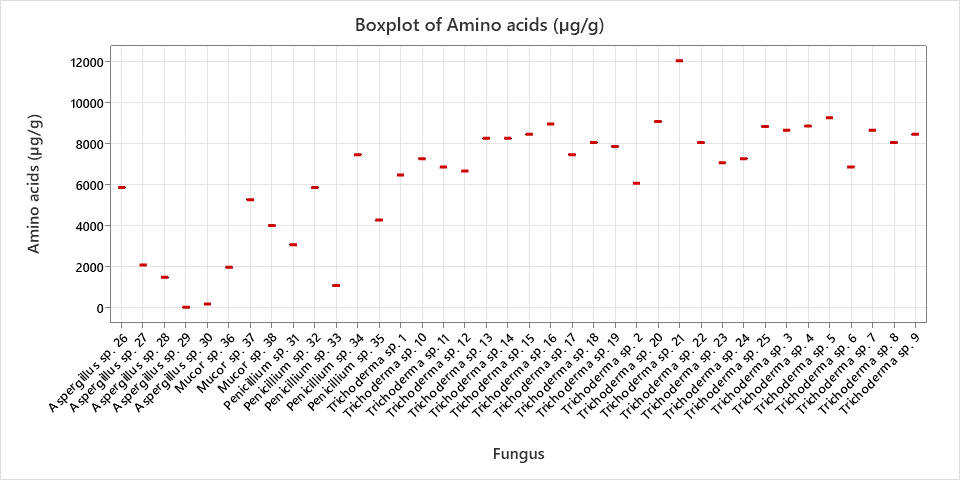


**Figure S1.** Boxplots of enzymatic activity and total free amino acids production of the isolated fungi on semi-solid-state fermentation medium, containing residue of common bean biomass as substrate.

**Molecular Identification**

**Analysis of the ITS region**

**Supplementary Table S1**. Identification of *Trichoderma* sp. WNZ-21 (OR857252.1) based on the ITS region and BLAST analysis.

| **Scientific name** | **Query cover** | **E value** | **Percentage of identity** | **Accession** |
| --- | --- | --- | --- | --- |
| *Trichoderma asperellum* | 100 | 0 | 97.88 | MF632083.1 |
| *Trichoderma asperelloides* | 100 | 0 | 94.62 | MK714898.1 |
| *Trichoderma* sp. | 100 | 0 | 94.62 | ON951840.1 |
| *Trichoderma* sp. | 100 | 0 | 94.42 | MK424131.1 |
| *Trichoderma asperellum* | 100 | 0 | 94.42 | MH598841.1 |
| *Trichoderma asperellum* | 100 | 0 | 94.43 | MF767444.1 |
| *Trichoderma* sp. | 100 | 0 | 94.42 | KY582099.1 |
| *Trichoderma asperellum* | 100 | 0 | 94.43 | KP263665.1 |
| *Trichoderma asperellum* | 100 | 0 | 94.43 | OM681183.1 |
| *Trichoderma asperellum* | 100 | 0 | 94.43 | JQ272474.1 |
| *Trichoderma asperellum* | 100 | 0 | 94.42 | MT529873.1 |
| *Trichoderma asperellum* | 100 | 0 | 94.42 | MT529846.1 |

**Analysis of the *tef1* gene**

**Supplementary Table S2.** Comparison of the *tef1* gene sequences of *Trichoderma asperellum* WNZ-21 (PP069312) with the other related sequences retrieved from the GenBank database.

| Scientific name | Accession | Query cover | E-value | Similarity (%) |
| --- | --- | --- | --- | --- |
| *Trichoderma asperellum* | MK439946.1 | 100% | 0 | 100 |
| *Trichoderma asperellum* | MK439944.1 | 100% | 0 | 100 |
| *Trichoderma asperellum* | MG595724.1 | 100% | 0 | 100 |
| *Trichoderma asperellum* | MG595723.1 | 100% | 0 | 100 |
| *Trichoderma asperellum* | MG595722.1 | 100% | 0 | 100 |
| *Trichoderma asperellum* | MG595721.1 | 100% | 0 | 100 |
| *Trichoderma asperellum* | MG595720.1 | 100% | 0 | 100 |
| *Trichoderma asperellum* | MG595719.1 | 100% | 0 | 100 |
| *Trichoderma asperellum* | MG595718.1 | 100% | 0 | 100 |
| *Trichoderma pseudoasperelloides* | MK775504.1 | 100% | 0 | 99.22 |
| *Trichoderma dingleyae* | OP962018.1 | 99% | 0 | 99.21 |
| *Trichoderma samuelsii* | MH746778.1 | 100% | 0 | 97.65 |
| *Trichoderma* sp. | MH606229.1 | 100% | 0 | 97.65 |
| *Trichoderma viride* | MG692546.1 | 100% | 0 | 97.65 |
| *Trichoderma koningiopsis* | OR687629.1 | 100% | 0 | 97.65 |
| *Trichoderma hispanicum* | JN715659.1 | 100% | 0 | 97.65 |
| *Trichoderma samuelsii* | JN715654.1 | 100% | 0 | 97.65 |

**Analysis of Trichoderma sp. WNZ-21 filtrate**

**Supplementary Table S3**. Chemical structure of the GC-MS compounds

| **No** | **Compound name** | **Chemical structure** |
| --- | --- | --- |
| 1 | (2S,3R)-3-[(4E,7E)-Nona-4,7-dienoyl]-N,N-bis(trimethylsilyl)oxirane-2-carboxamide | 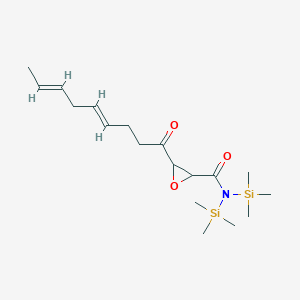 |
| 2 | Silane, [(1-methoxy-1,3-propanediyl)bis(oxy)]bis[trimethyl- | 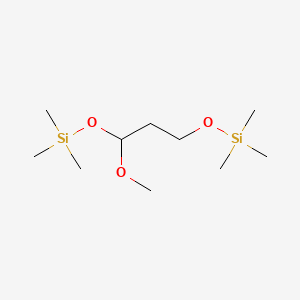 |
| 3 | Glycerol, 3TMS derivative | 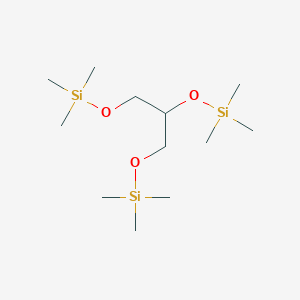 |
| 4 | Erythro-Pentonic acid, 2-deoxy-3,4,5-tris-O-(trimethylsilyl)-, trimethylsilyl ester | 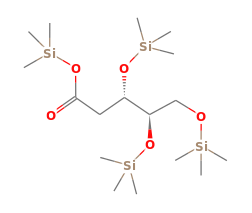 |
| 5 | Tartaric acid, 4TMS derivative | 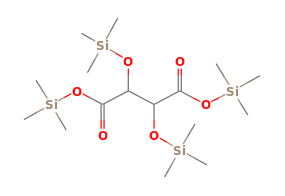 |
| 6 | D-Glucitol, 6TMS | 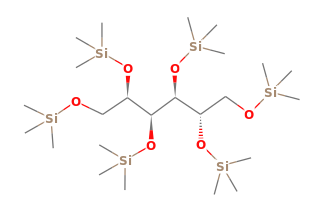 |
| 7 | Terephthalic acid, 2TMS derivative | 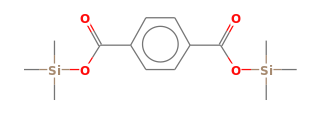 |
| 8 | Azelaic acid, 2TMS derivative | 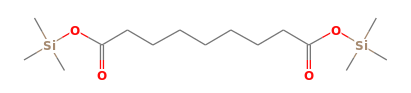 |
| 9 | D-Pinitol, pentakis(trimethylsilyl) ether | 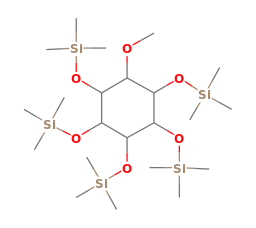 |
| 10 | Cyclopropanedodecanoic acid, 2-octyl-, methyl ester | 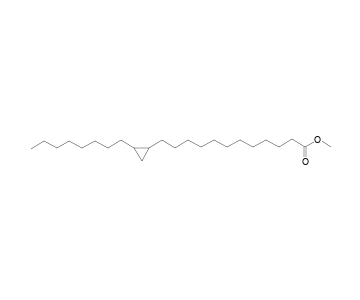 |
| 11 | D-Mannitol, 6TMS | 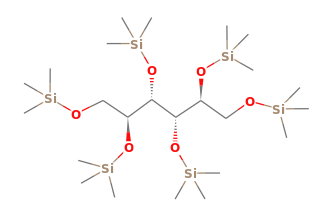 |
| 12 | Palmitic Acid, TMS derivative | 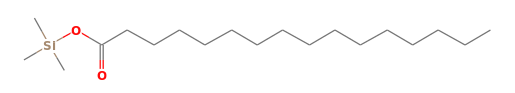 |
| 13 | 9-Octadecenoic acid (Z)-, 2-hydroxy-1-(hydroxymethyl)ethyl ester | 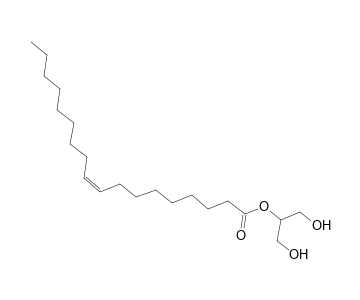 |
| 14 | Stearic acid, TMS derivative | 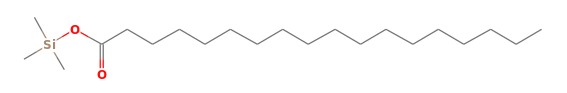 |
| 15 | Behenic acid, TMS derivative | 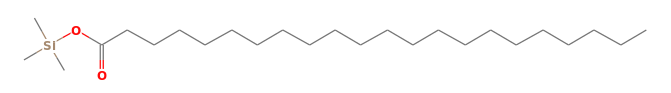 |
| 16 | Lignoceric acid, TMS derivative | 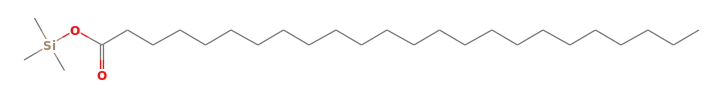 |
| 17 | Silane, diethylheptyloxyoctadecyloxy- | 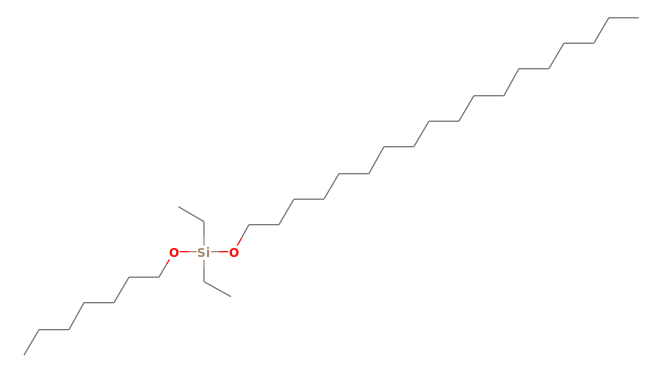 |


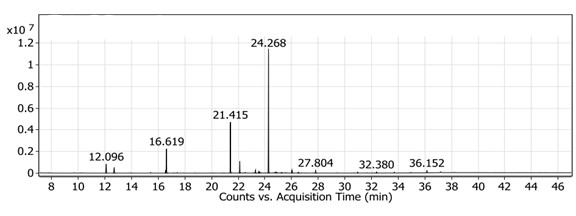


**Supplementary Figure S2**. GC-MS analysis of *Trichoderma* *asperellum* WNZ-21 filtrate


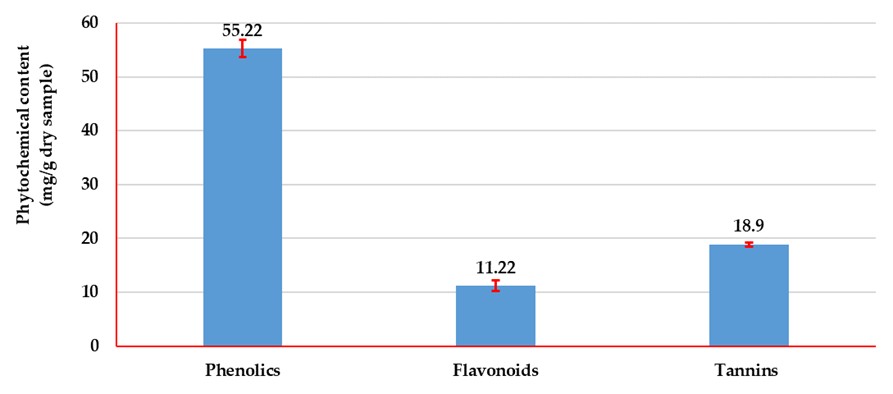


**Figure S3**. The level of phytochemical contents fungal filtrate of *T.* *asperellum* WNZ-21.


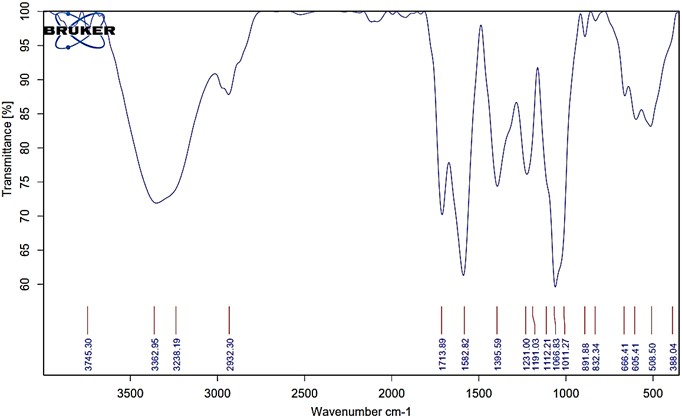


**Figure S4.** FT-IR spectrum of *Trichoderma* *asperellum* WNZ-21 filtrate.

**FT-IR Spectrum**

**Supplementary Table S4**. FT-IR Spectrum of *Trichoderma* *asperellum* WNZ-21 Fungal Filtrate.

| Wavenumber (cm^-1^) | Functional Group | Interpretation | Potential Components |
| --- | --- | --- | --- |
| 3362, 3238 | NH stretches | Primary and secondary amines (amino acids, other nitrogenous compounds) | Lysine, arginine, glutamic acid, alanine, ammonia, methylamine |
| 2932 | CH stretches | Alkyl groups (amino acid chains, other organic molecules) | Leucine, isoleucine, valine, fatty acids, lipids |
| 1713, 1645 | C=O stretches | Carboxylic acid groups and carbonyl groups (amino acids, peptides, other metabolites) | Aspartic acid, glutamic acid, asparagine, glutamine, ketones, organic acids |
| 1395, 1231 | C-N stretches | Amino functional groups (amino acids, proteins) | Arginine, lysine, histidine, cysteine, peptides, protein backbone |
| 1191, 1112, 1066, 1011 | Phosphates, sulfates | Nucleotides (RNA, DNA), phospholipids, sulfated polysaccharides (heparin, chondroitin sulfate) | - |
| 891 | Aromatic C-H bending vibrations | Potential aromatic rings | Phenylalanine, tyrosine, tryptophan, aromatic metabolites, benzene derivatives |
| 832 | C-O stretches | Carbohydrates or polysaccharides | Glucose, mannose, galactose, chitin, glucans |
| 666, 605 | Fingerprint region | Further investigation is needed (potential sulfates, aromatic compounds) | Sulfates in polysaccharides or proteins, specific aromatic ring structures |
| 508 | Fingerprint region | Further investigation needed (specific to molecular structure) | May reveal specific sugar types, protein secondary structure, unique components |

**Biological Activity**

**Cytotoxic activity of the fungal filtrate**

| 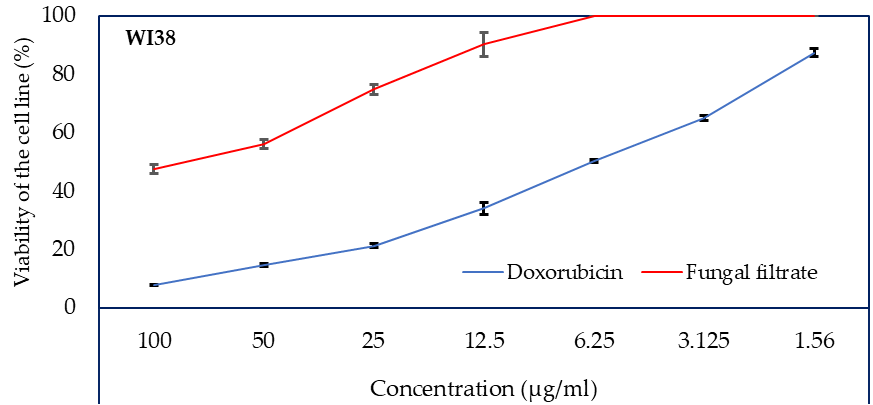 |
| --- |
| 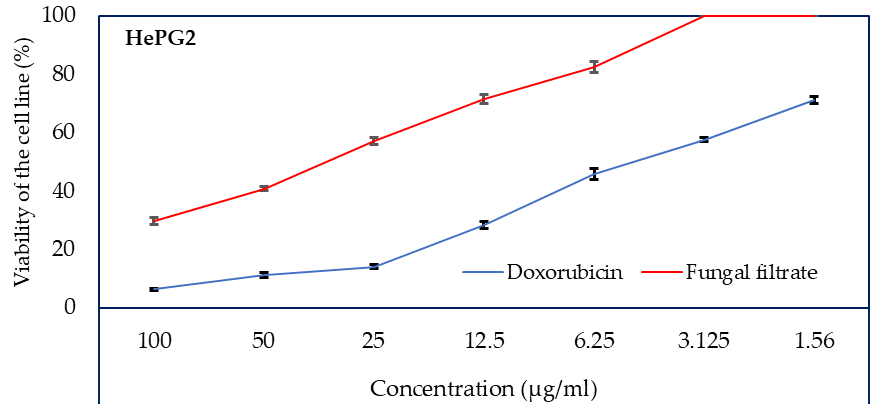 |
| 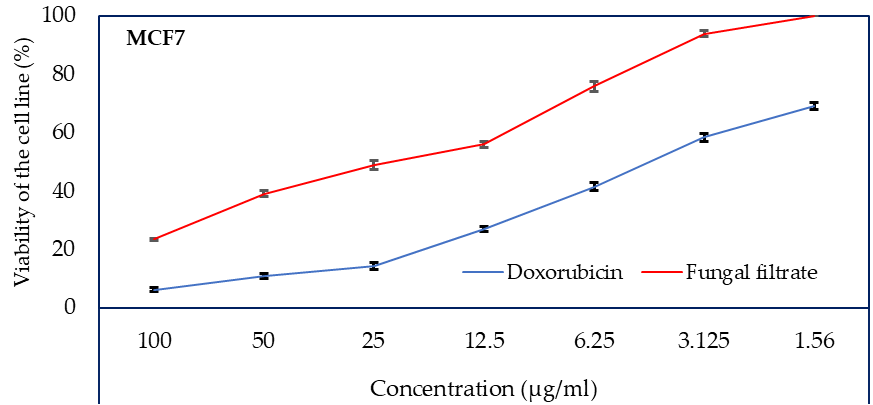 |

**Supplementary** **Figure S5**. Relative viability of the cell lines of human lung fibroblast (WI38), hepatocellular carcinoma (HePG2), and mammary gland breast cancer (MCF7), as affected by *Trichoderma* *asperellum* WNZ-21 fungal filtrate and standard doxorubicin drug.

**References**

Asis, A., Shahriar, S. A., Naher, L., Saallah, S., Fatihah, H. N. N., Kumar, V., & Siddiquee, S. (2021). Identification patterns of Trichoderma strains using morphological characteristics, phylogenetic analyses and lignocellulolytic activities. *Mol Biol Rep*, *48*(4), 3285-3301. <https://doi.org/10.1007/s11033-021-06321-0>

de Hoog, G. S., Guarro, J., Gené, J., & Figueras, M. (2005). Atlas of clinical fungi.

Diba, K., Kordbacheh, P., Mirhendi, S., Rezaie, S., & Mahmoudi, M. (2007). Identification of Aspergillus species using morphological characteristics. *Pakistan journal of medical sciences*, *23*(6), 867.

Domsch, K. H., Gams, W., & Anderson, T.-H. (1980). *Compendium of soil fungi. Volume 1*. Academic Press (London) Ltd.

Samson, R. A., & Pitt, J. I. (2000). *Integration of modern taxonomic methods for Penicillium and Aspergillus classification*. CRC Press.

Samson, R. A., Visagie, C. M., Houbraken, J., Hong, S. B., Hubka, V., Klaassen, C. H., Perrone, G., Seifert, K. A., Susca, A., Tanney, J. B., Varga, J., Kocsube, S., Szigeti, G., Yaguchi, T., & Frisvad, J. C. (2014). Phylogeny, identification and nomenclature of the genus Aspergillus. *Stud Mycol*, *78*(1), 141-173. <https://doi.org/10.1016/j.simyco.2014.07.004>

Siddiquee, S., & Siddiquee, S. (2017). Morphology-based characterization of Trichoderma species. *Practical Handbook of the Biology and Molecular Diversity of Trichoderma Species from Tropical Regions*, 41-73. <https://doi.org/https://doi.org/10.1007/978-3-319-64946-7_4>

St-Germain, G., & Summerbell, R. (2003). Identifying filamentous fungi: a clinical laboratory handbook. In: SciELO Brasil.

Sutton, D. A., Fothergill, A. W., & Rinaldi, M. G. (1998). *Guide to clinically significant fungi*. Williams & Wilkins.
